# Plasmid Vectors and Molecular Building Blocks for the Development of Genetic Manipulation Tools for *Trypanosoma cruzi*

**DOI:** 10.1371/journal.pone.0080217

**Published:** 2013-10-24

**Authors:** León A. Bouvier, María de los Milagros Cámara, Gaspar E. Canepa, Mariana R. Miranda, Claudio A. Pereira

**Affiliations:** Laboratorio de Biología Molecular de *Trypanosoma cruzi* (LBMTC), Instituto de Investigaciones Médicas “Alfredo Lanari”, Universidad de Buenos Aires and CONICET, Buenos Aires, Argentina; Federal University of São Paulo, Brazil

## Abstract

The post genomic era revealed the need for developing better performing, easier to use and more sophisticated genetic manipulation tools for the study of *Trypanosoma cruzi*, the etiological agent of Chagas disease. In this work a series of plasmids that allow genetic manipulation of this protozoan parasite were developed. First of all we focused on useful tools to establish selection strategies for different strains and which can be employed as expression vectors. On the other hand molecular building blocks in the form of diverse selectable markers, modifiable fluorescent protein and epitope-tag coding sequences were produced. Both types of modules were harboured in backbone molecules conceived to offer multiple construction and sub-cloning strategies. These can be used to confer new properties to already available genetic manipulation tools or as starting points for whole novel designs. The performance of each plasmid and building block was determined independently. For illustration purposes, some simple direct practical applications were conducted.

## Introduction

Genetic manipulation has played an important role in the understanding of the biology of *Trypanosoma cruzi*. In men, infection with this protozoan parasite produces a condition known as Chagas disease which affects at least seven million people [1]. During its life cycle *Trypanosoma cruzi*, presents four main developmental stages; replicative epimastigotes and metacyclic trypomastigotes which are found in the insect vector while the bloodstream trypomastigote and proliferative intracellular amastigote reside in the vertebrate host [2]. It has been shown that this parasite has a complex population structure with a wide genetic and biochemical diversity among different strains and natural isolates, accounting for their particular features regarding eco-epidemiology, pathogenicity and biology [3]. Consequently there is growing interest in employing genetic manipulation techniques in order to gain a better understanding of its biology.

As in other trypanosomatids, the diploid genome is organized in policistronic gene clusters with tens-to-hundreds of intron less protein coding sequences arranged sequentially on the same strand and held apart by intergenic sequences (IS) [4]. These clusters are transcribed by RNA polymerase II into policistronic transcripts and mRNA maturation for each gene involves trans-splicing reactions. Centered on a transcribed IS, the trans-spliceosomal complex is responsible for the polyadenylation of the upstream gene and the addition of the spliced leader (SL), a short capped RNA, to the 5´ end of the downstream gene in a concerted fashion [5]. Furthermore, ISs are the source of untranslated regions (UTR) which determine mRNA stability and steady state levels, as well as translation efficiencies [6]. Consequently, developmental stage specific or constitutive gene expression profiles are mostly defined by ISs in a posttranscriptional fashion.

Standard genetic manipulation of *Trypanosoma cruzi* is performed with epimastigotes under axenic culture conditions. Basic genetic manipulation tools correspond to episomal expression vectors (EEVs). Mimicking the particular genomic organization of these organisms, the transgenes are placed in the same orientation between ISs derived from constitutive (housekeeping) genes. Analogously, antibiotic resistance genes flanked by ISs make up trypanosome selectable markers (SMs). Closed circular plasmids containing such an alternation of regulatory intergenic and coding sequences can be stably maintained as autonomous replicating extrachromosomal molecules [7]. In this type of construct transcription takes place even in the absence of specific promoter sequences. The inclusion of RNA polymerase I ribosomal promoters into EEVs, significantly increases transgene expression [8] and confers integrative properties, since such circular constructs have been shown to recombine into ribosomal loci [9]. The genetic manipulation tools most frequently used are expression vectors that date to the pre-genomic era. In order to take advantage of the available sequence data, genetic systems based on more efficient and less time consuming construction technologies were developed for the generation of targeted gene replacement vectors (TGRVs) [10] and the expression of fusion proteins [11]. In some cases, attention was paid on producing flexible tools capable of exchanging different constituting elements [11], which is a property absent from most of the existing designs. With TGRVs it has been possible to generate single and double null mutant *T. cruzi* strains [10]. Since this trypanosome lacks RNA interference pathways [12] TGRV based strategies constitute, to date, the only available alternative to specific eliminate a gene´s expression product. 

This work was focused on the production of two types of molecular building blocks. The first set of modules correspond to general purpose SMs. The importance of these molecules resides in that many methodologies, which imply expression of multiple transgenes, like inducible expression systems or the generation of null mutant strains, require successive or simultaneous transfection and selection steps. The second group of building blocks correspond to coding sequences for traceable elements like fluorescent proteins and epitope tags. These allow the detection of fused proteins of interest and therefore the production of specifically raised antibodies can be avoided. In both cases the design maximizes the cloning and modification possibilities thus allowing a broad range of applications, from conferring new features to already available genetic manipulation tools, the development of novel manipulation constructs to the study of particular genes of interest. 

## Materials and Methods

### Parasites

*Trypanosoma cruzi* epimastigotes of the MJ-Levin, Dm28c and Y strains were grown at 28 °C in 25 cm^2^ culture flasks containing 5 mL of LIT medium (devised by Yeager [13]) supplemented with 10% fetal bovine serum, 100 U/mL penicillin and 100 µg/mL streptomycin. The parental wild type (WT) as well as all transgenic selected cell lines were routinely subcultured with weekly passages, for this, fresh medium was supplemented with the respective antibiotics to the indicated concentrations and inoculated to 1x10^6^ parasites/mL. Cells were counted with a hemocytometer. 

### Plasmid construction

DNA ligase, polynucleotide kinase, Klenow DNA polymerase and all restriction enzymes, employed were purchased from New England Biolabs (NEB) except Csp45I and shrimp alkaline phosphatase which were obtained from Promega. Pfu and Taq DNA polymerases were respectively purchased from Fermentas and Sigma. The oligonucleotides used in this work are listed in Methods S1 and were ordered to Sigma Life Science. For the construction of pTREXL, pTEXL, p*Tc*R and pDIY vectors along with their respective accession numbers see Methods S1. For the construction of TGRV and EEV for *TcADK4* a 2.7 kbp genomic fragment was PCR-amplified with primers 4F and 4R and cloned into pGEM-T Easy (Promega). A clone with the fragment inserted in the opposite orientation relative to the direction of T7 promoter transcription was isolated and designated pGEM-T Easy L4. A 5.1 kbp fragment purified from this plasmid after digestion with BamHI and Csp45I was ligated to Hyg and Neo SMs derived respectively from p*Tc*R-HG Hyg - and p*Tc*R-GA Neo - by digestion with the same enzymes. The resulting vectors were designated pL4-HG Hyg and pL4-GA Neo. Similarly pL4X-HG Neo was produced inserting the Neo SM of p*Tc*R-HG Neo - into pGEM-T Easy L4 employing the NsiI and SalI restriction enzyme sites present in both molecules. For the GFP tagged EEV, first a stop codon was generated immediately downstream from the eGFP gene of pDIY-eG by digestion with HindIII, treatment with Klenow DNA polymerase and blunt end religation. Employing BamHI and Csp45I restriction enzyme sites the fluorescent protein gene of this plasmid was inserted into pGEM-T Easy L4, replacing a section of *TcADK4*, and thus obtaining the intermediate pL4X-GFP. A 2.4 kbp NsiI-BamHI segment comprising HG-Neo, intergenic and remaining coding sequences of pL4X-HG Neo were inserted into pL4X-GFP generating pL4X-GFP HG Neo. 

### Plasmid preparation for transfection

For transfections with supercoiled molecules, plasmids were recovered from 10 mL *E. coli* cultures grown to saturation in LB medium [14]. After a standard alkaline lysis [15] supernatants were incubated for 1 h at 37 °C with 25 µg/mL RNase A (QIAGEN) and extracted twice with chloroform:isoamyl alcohol (24:1, v/v). DNA was precipitated by addition of an equal volume of isopropanol and pellets were combined. For transfections with linearized plasmids, these were purified from an equivalent bacterial culture with a single column of the GenElute™ HP Plasmid Miniprep Kit (Sigma). DNA was eluted with 100 µL of 1X restriction enzyme buffer and overnight digested with 20 units of NotI. The linearized plasmid was precipitated by addition of 10 µL of 3 M Potassium acetate pH 4.8 and 250 µL of 100% ethanol. Pellets obtained by both methodologies were washed with 70% ethanol, resuspended in 50 µL of TE (10 mM Tris-HCl, pH 8; 1 mM EDTA) and stored at -20 °C. Each sample which corresponded to approximately 20 µg of DNA was used for a single transfection. In order to diminish the chance of culture contamination, prior to transfection, DNA was heated at 80 °C for 10 minutes. All plasmids were transfected as circular molecules except those intended for targeted replacement of the *TcADK4* gene which were linearized with NotI. 

### Parasite transfections and selection

Epimastigotes grown to a density of 2.5 x 10^7^ cells/mL were harvested by centrifugation at 3,000 x g for 10 minutes and washed once with electroporation buffer (PBS: 137 mM NaCl; 2,7 mM KCl; 10 mM Na_2_HPO_4_; 2 mM KH_2_PO_4_; pH 7.4 supplemented with 0,5 mM MgCl; 0,1 mM CaCl_2_). Between 2 x 10^8^ and 5 x 10^8^ cells were resuspended in 350 µL of the same buffer and placed in a 0.2 cm gap electroporation cuvette containing the previously prepared DNA or TE for mock transfected parasites. The suspension was subjected to a single exponential-decay discharge of 400 V and 500 µF (τ^≈^5 ms) administered with a Gene Pulser Xcell system (Bio-Rad) and after electroporation the cells were transferred to 5 mL of medium. Antibiotics were added to the initial indicated concentrations 24 h post-transfection, and after an additional 24 h period, cell cultures were passaged at a 1:10 dilution maintaining the antibiotic concentration. Cellular morphologies and culture densities were monitored periodically by microscopic inspection for 15 days. Cultures were further passaged only if, after this period, at least a few motile cells with normal morphology were detected per field. In order to avoid low cellular density associated stress, during the first passages transgenic lines were subcultured at high densities (diluted 1:5) with unmodified antibiotic concentrations while fast recovering cultures were diluted up to 1:100 and challenged with increasing antibiotic concentrations. Further experiments were carried out with selected parasite populations. Antibiotics: blasticidin S and phleomycin were obtained from Invivogen, hygromycin B was purchased from Invitrogen and G418 from Calbiochem.

### Parasite DNA purification and analysis

Approximately 2 x 10^7^ parasites were harvested by centrifugation at 3,000 x g for 10 minutes, washed once with PBS and resuspended in 1 mL of lysis buffer (10 mM Tris-HCl, pH 7.5; 100 mM EDTA; 0.1% SDS). The samples were treated with 0.5 mL of phenol: chloroform:isoamyl alcohol (25:24:1, v/v) and the aqueous phase was further extracted once with chloroform:isoamyl alcohol (24:1, v/v). DNA was precipitated by addition of an equal volume of isopropanol and spooled up on a glass rod. DNA was washed with 70% ethanol, resuspended in 50 µL of 10 mM Tris-HCl, pH 8 containing 25 µg/mL RNase A (QIAGEN) and stored at 4 °C. For the detection of BSD, ble, Hyg and Neo resistance genes and the presence of *TcADK4* recombinant loci were amplified by PCR with the primers listed in Methods S1.

### Florescence microscopy

Epimastigotes were washed once, resuspended to a density of 10^7^ cells/mL with PBS, and allowed to settle on poly-L-lysine coated microscope slides for 10 minutes. Cells were fixed with a solution of 4% paraformaldehyde supplemented PBS for 10 minutes. Slides were washed with PBS, mounted with anti-fade mounting medium (PBS, 90% glycerol, 0.2% n-propyl gallate, 1.5 µg/mL DAPI: 4’,6 diamidino-2-phenylindole) and inspected with an Olympus BX51 fluorescence microscope. 

### Western-Blot assays

Parasites were washed once with PBS, resuspended in Laemmli sample buffer [16] (60 mM Tris-Cl pH 6.8, 2% SDS, 10% glycerol, 5% β-mercaptoethanol, 0.01% bromophenol blue) to a density of 2 x 10^8^ cells/mL and boiled for 10 minutes. Samples were resolved by SDS-PAGE. Western Blots were performed using total *T. cruzi* extracts fractioned by electrophoresis in polyacrylamide denaturing gels and transferred to polyvinylidene fluoride (PVDF) membranes. The PVDF membranes were treated for 1 h with 5% non-fat dry milk in PBS and then incubated with the primary antibody ON, anti-GFP rabbit polyclonal (1:5000) (Invitrogen), anti-PAR2 mouse monoclonal (1:2000), anti-FLAG clone M2 mouse monoclonal (1:4000) (Sigma), anti-HA clone 3F10 rat monoclonal (1:4000) (Roche), YL1/2 rat monoclonal (1:4000) (Chemicon) and anti-ADK4 mouse polyclonal serum (1:2000). Membranes were washed and incubated with the corresponding horseradish peroxidase-conjugated secondary antibody, horse anti-mouse IgG (1:5000) (Vector Labs), goat anti-rabbit IgG (1:5000) (Vector Labs) and goat anti-rat IgG (1:4000) (Jackson Immuno Research) for two hours. Detection was done with SuperSignal West Pico Chemiluminescent Substrate (Pierce) according to manufacturer´s instructions.

### Bioinformatics

Sequence analysis was performed with EMBOSS [17] and plasmids were designed with Vector-NTI Advance 10 (Invitrogen). Custom made analysis scripts were programed in Perl (www.perl.org). For the MCSs of pMCS, eight compatible end restriction enzyme site pairs (EcoRI, MfeI; Acc65I, BsrGI; NcoI, BspHI; SalI, XhoI; PstI, NsiI; BglII, BamHI; XbaI, NheI and Csp45I, ClaI) were chosen and the minimum length overlapping MCSs for all possible 8-member sets considering all permutations were obtained with an exhaustive search algorithm (search space = 2^8^ x 8!). Shortest sequences with sites overlapping *dam* methylase motifs were selected and HindIII, NotI, EcoRV and PvuII recognition sites were manually added. For gene restriction analysis, suitable restriction enzyme sites were identified in The Restriction Enzyme Database (rebase.neb.com) and predicted coding sequence data sets (version 3.2) from *T. cruzi* CL Brener, *T. brucei* TREU 927, *T. brucei* Lister 427 and *L. major* Friedlin strains were obtained from TriTrypDB [18] (tritrypdb.org). Perl scripts specific for p*Tc*R and pDIY vectors are available and can be adapted for the analysis of other sequences. 

## Results and Discussion

### Selectable marker development

The first objective of this work was the development of novel SMs for different antibiotic resistance genes. The design was mainly focused on providing multiple alternatives for transferring the SMs to other genetic constructs. Since such a development would require many genetic engineering procedures, it was performed in a stepwise fashion. The first step consisted in the construction of the SMs in the context of green fluorescent protein (GFP) expression vectors. These would allow to easily verify if the SMs performed as expected (i. e. were capable of conferring resistance to their respective antibiotics). Furthermore these by-product plasmids could alternatively be employed as general purpose expression vectors and additionally be helpful in devising and improving selection schemes according to the local culture and transfection methodologies employed by each group. 

**Figure 1:**
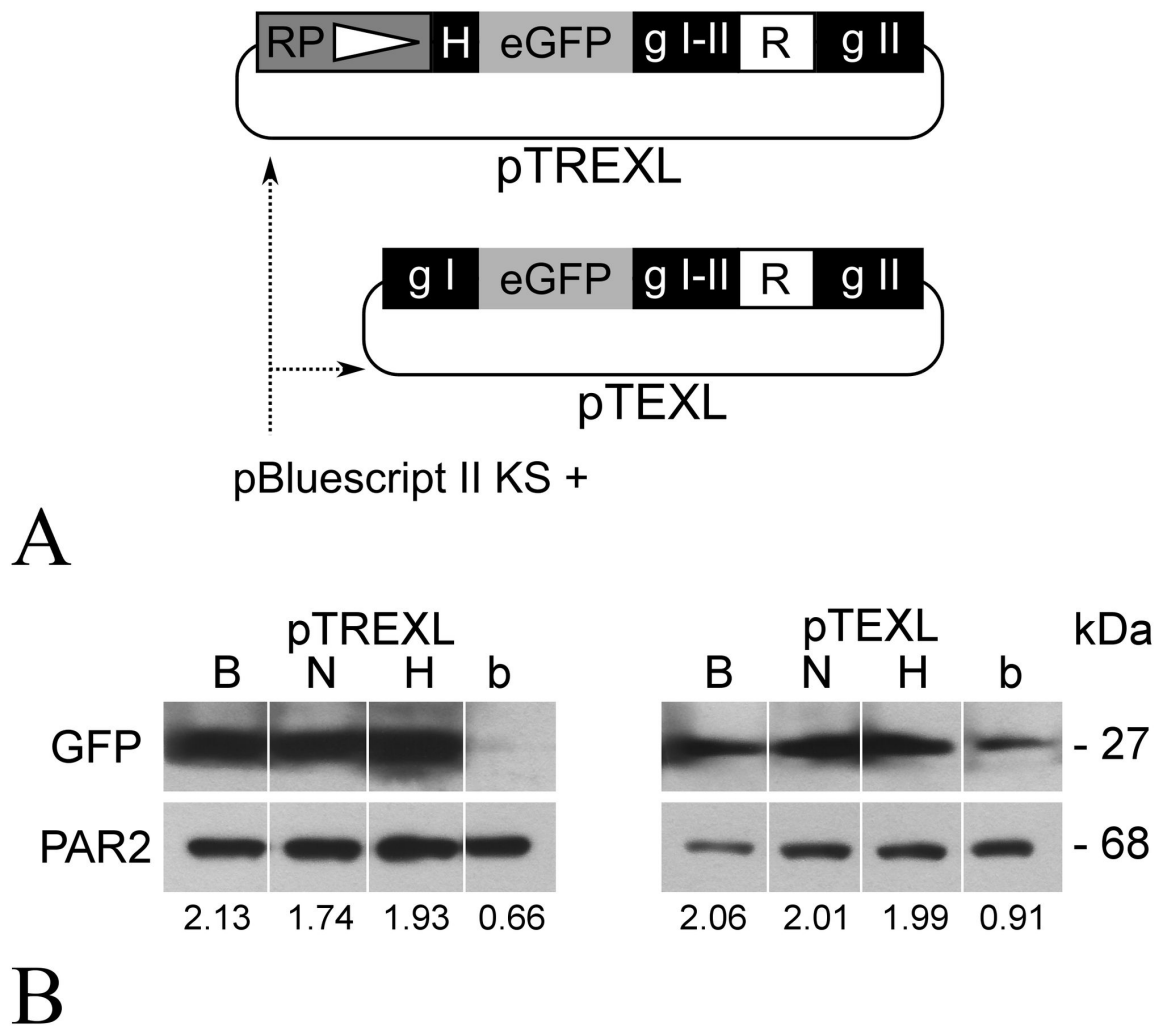
Expression vectors pTREXL and pTEXL. (A) Schematic maps. The white box labelled R represents any of the four antibiotic resistance genes (BSD, blasticidin S deaminase; ble, bleomicin antibiotic family resistance gene; Hyg, hygromycin B phosphotransferase; Neo, aminoglycoside phosphotransferase). Black boxes are RNA processing sequences, the ones labelled g I, g I-II and I g II correspond to ISs upstream, relative to and downstream from the g*GAPDH* tandem copy genes respectively. H contains the ribosomal protein P2β splice acceptor site and the white triangle inside the grey box represents the ARN pol I ribosomal promoter (RP). The enhanced green fluorescent protein coding sequence is shown as a grey box labelled eGFP. The pBluescript II KS + phagemid used as backbone is indicated by the dotted arrows. For an extended restriction map see Vector Information S1. (B) Western blot analysis of whole cell extracts derived from pTREXL (left) and pTEXL (right) transfected epimastigote lines. Lanes labelled B, N, H and b correspond to selected cultures maintained at least for two months with 100 µg/mL, 500 µg/mL, 1 mg/mL and 500 µg/mL (pTREXL) or 50 µg/mL, 200 µg/mL, 500 µg/mL and 250 µg/mL (pTEXL) of blasticidin S, G418, hygromicin B and Phleomycin respectively. After electroblotting membranes where stained with anti-GFP rabbit polyclonal antibody (top) or anti-PAR2 mouse monoclonal antibody (bottom). Each sample corresponds to 4·10^6^ cells. Below the lanes, eGFP expression levels relative to those of PAR2 are indicated.

The first four SMs constructed were based on the one present in the pTEX [7] expression vector with *gGAPDH* ISs flanking phleomycin (ble), blasticidin S (BSD), hygromycin B (Hyg) or G418 (Neo) resistance genes. The addition of a ribosomal promoter and the IS of *TcP2β* genes (HX1) upstream the eGFP coding sequence (Clontech) to each SM yielded a pTREX [19] like (pTREXL) configuration (Figure 1A). The four resulting expression vectors were transfected into the recently typified MJ-Levin strain (Dr. A. Schijman personal communication) and after electroporation, cells were subjected to selection with initial antibiotic concentrations conservatively chosen according to the available literature (Table 1). The initial selection period lasted approximately a month after which a large proportion (^≈^ 80%) of fluorescent cells could be detected in all cultures except in the ones selected with phleomycin. Noteworthy the growth rate of these selected lines were indistinguishable from those of the parental un-transfected strain. To evaluate the performance of the pTREXL vectors in different strains, the transfections were repeated using the Dm28c and Y strain in addition to the MJ-Levin strain. Initial antibiotic concentrations employed are detailed in Vector Information S1. Although viable cultures could be obtained for the transfected MJ-Levin and Dm28c strain, none of the pTREXL vectors seemed capable of conferring resistance to the Y strain, since all the cultures relative to it perished. On the other hand, most of the viable MJ-Levin and Dm28c strain cells were fluorescent except those transfected with pTREXL-ble. Noteworthy, after this initial selection scheme, the resulting cultures were challenged with concentration of antibiotics lengthy exceeding the IC50s (Table 1) without any effect on viability.

**Table 1:** Antibiotic concentrations (µg/mL) employed in this work.

**Marker**	**Drug**	**IC50 - Strain**		**Construct**				**Reference**
		**Antibiotic concentrations (µg/mL)**						
		**MJ**	**Y**	**Mock**	**pTREXL**	**pTEXL**	**p*Tc*R**	
ble	Phleomycin	52.8	73.3	100	100 → 500	250	250 → 500	100 [35] - 500 [36]
BSD	Blasticidin S	36.3	22.8	10 - 40	10 → 100	50	10 → 50	10 [37]
Hyg	Hygromycin B	347.2	277.5	250	250 → 1000	250 → 500	200 → 1000	100 [23] - 600 [10]
Neo	Geneticin (G418)	121.5	113.0	100	500	100 → 200	100 → 500	100 [7] - 500 [36]

The IC50 values for each drug were obtained for the MJ-Levin and Y strain. The concentration used during the selection of cells transfected with the vector developed in this work as well as those for mock transfected parasites are indicated. Reported lowest and highest concentrations employed by other authors have been included for comparative reasons. Any concentration inside a given interval is expressed with a dash while an arrow indicates a gradual increase in concentration along successive culture passages.

The failure of the SMs contained in the pTREXL vectors to confer antibiotic resistance to the Y strain suggested a possible incompatibility due to the harboured ribosomal promoter sequence. Additionally the vectors with resistance genes for antibiotics that target translation (blasticidin S, hygromicin B and G418) all yielded fluorescent cells when selected in contrast to phleomycin that targets and damages DNA. For this reasons the four pTREXL plasmids were modified into more general EEVs replacing the ribosomal promoter sequence and the HX1 region with the IS upstream of the *gGAPDH I* gene rendering a pTEX [7] like configuration (Figure 1A). These EEVs were transfectetd, into the three *T. cruzi* strains used for pTREXL evaluation. Initial antibiotic concentrations employed are detailed in Vector Information S1. Selection of these cultures took considerably shorter, between 15 and 20 days. The predominant viable cells were fluorescent irrespective of strain and construct. As expected for EEVs there was a marked heterogeneity regarding GFP expression levels between different cells from the same culture (see Results S1). 

Once selected, parasites were kept in culture with regular weekly passages under selective conditions with the highest antibiotics concentrations in Table 1 for at least two months. Finally cell extracts were assayed for GFP expression by Western-Blot (Figure 1B). The absence of expression in cells transfected with pTREXL-ble could be confirmed. In the remaining extracts GFP expression levels were comparable. Western Blot band intensity was quantified and relativized to PAR2 expression revealing similar expression levels for all the tested plasmids, indicating similar expression efficiencies.

Transient transfection and selection efficiencies of the pTREXL-Neo and pTEXL-Neo expression plasmids were equivalent from those obtained for the related eGFP harbouring original pTREX and pTEX expression vectors.

Considering host range and initial selection times, pTEXL vectors performed better than pTREXL equivalents. Nevertheless cultures transgenic for the latter had more homogeneous expression patterns. 

### Development of general purpose selectable markers: p*Tc*R vector series

**Figure 2:**
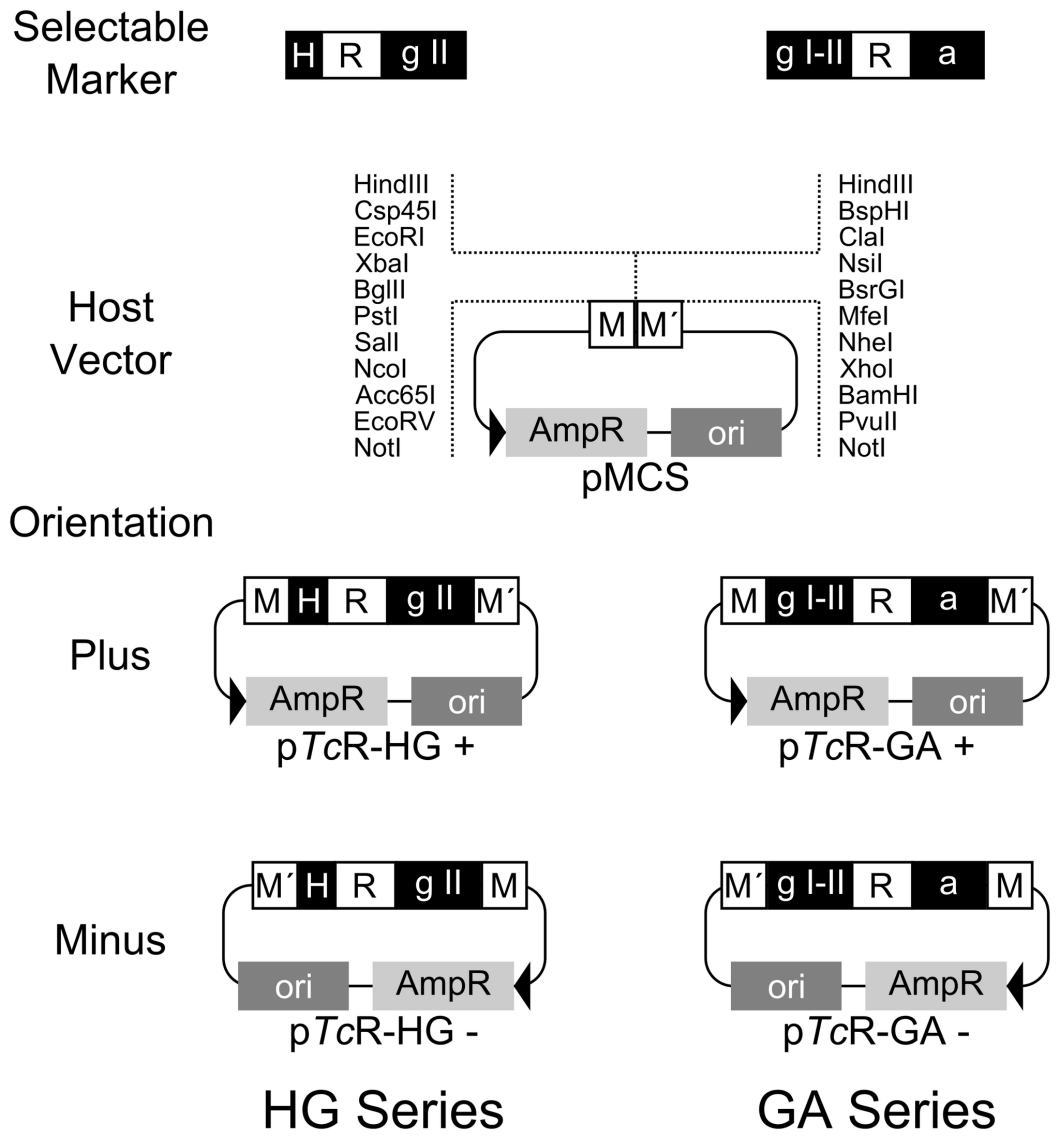
Schematic maps of p*Tc*R vector series. The HG and GA selectable marker series contain each of the four antibiotic resistance genes (BSD,ble,Hyg and Neo; white box R) between the HX1 (H) and gapdh II (g II) or gapdh I-II (g I-II) and actin (a) ISs (black boxes) respectively. The pMCS host vector is schematized below with the two multiple cloning sites (M and M´) the ampicillin resistance gene (AmpR) and replication origin (ori). The resulting p*Tc*R vectors obtained cloning each SM into pMCS in either orientation are shown below. For an extended restriction map of p*Tc*R vector series see Vector Information S1.

Having found that the SMs so far developed could confer resistance to different antibiotics in different *T. cruzi* strains, the next step towards a modular SM design was undertook. Employing the pTREXL vectors as starting point, the additional SM series HG and GA (Figure 2) were produced. In the latter the IS relative to the *actin 1* and *actin 2* tandem copy genes [20,21] was inserted downstream from the resistance genes. As a host vector for the different HG and GA SMs series the minimal, pUC derived, pMCS plasmid was constructed. The MCS in this vector was designed so that, upon SM insertion, the two resulting halves would share some symmetrical properties. Each side includes sites for rare-cutting and blunt end restriction enzymes. Additionally there are sites for eight compatible end restriction enzyme pairs, one on each side of the cloned SM. For every SM two p*Tc*R vectors were obtained, corresponding to the two possible orientations of insertion into pMCS (Figure 2). Given this design the SMs can be easily subcloned into any of 29 commercially available restriction enzyme sites irrespective of their order of occurrence and even if the destination molecule contains only one of these sites at the desired location. Additionally, for high-throughput methodologies, the p*Tc*R vectors can be used with the In-Fusion seamless cloning system [22] (Clontech) or easily converted to Gateway (Invitrogen) cloning compatible plasmids. In all, the number of potential destination plasmids for a p*Tc*R derived SM is very large. See Vector Information S1 for details on how to use In-Fusion or Gateway cloning with p*Tc*R vectors and for suggested SM transfer strategies to pTEX [7], pRIBOTEX [8], pTREX [19], pTcINDEX [23] and p*Tc*GW [11] expression vectors.

The most basic property the p*Tc*R vector series was expected to fulfill was the capacity to confer resistance to the respective antibiotics. Consequently epimastigote cells from the MJ-Levin and Y strains were transfected with each of the 16 members of the p*Tc*R vector series and subjected to selection with the initial antibiotic concentrations detailed in Vector Information S1. Depending on the antibiotic the initial selection period lasted between 18 and 28 days. The concentration of the different selective agents was gradually increased in successive passages and the parasites were kept in culture for at least 70 days. The accumulated dilution factor was approximately 10^-12^. At this point, it was considered that all the cell lines had successfully acquired the respective p*Tc*R encoded resistances. It was expected that the acquired resistence which exceeded in each case the IC50 for each antibiotic (Table 1) correlated with the presence of the coding DNA for each of the respective resistance genes. Therefore, from each final cellular culture, genomic DNA was obtained and the presence of p*Tc*R derived material was determined by PCR amplification of the different resistance genes. Expected amplification products were obtained for each culture irrespective of strain and vector (data not shown).

**Figure 3:**
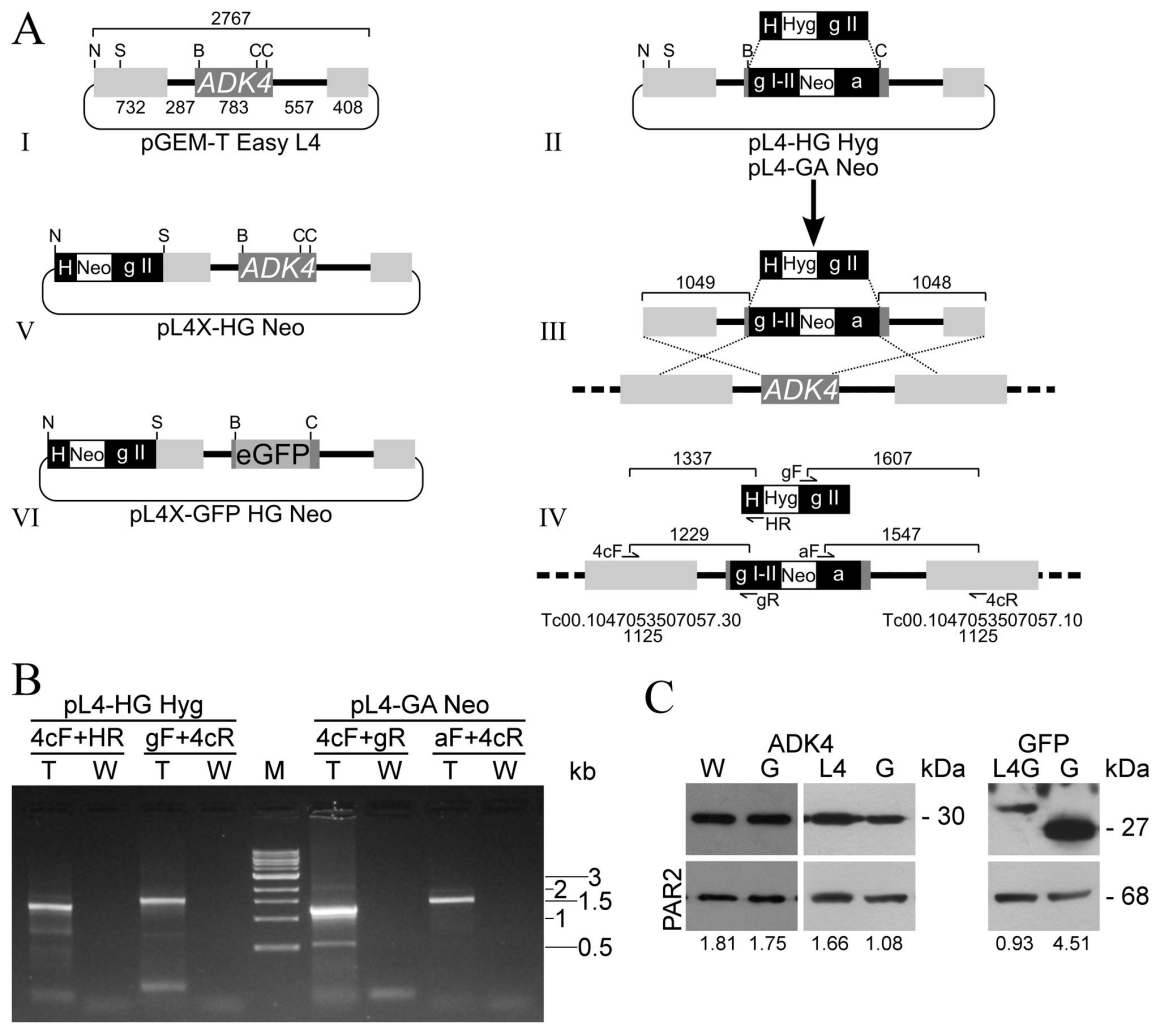
Application of developed building blocks in the construction of genetic manipulation vectors. (A) Diagram showing the TGRV and EEV constructed for the adenylate kinase 4 (TcADK4) gene. I, pGEM-T Easy with a cloned genomic fragment comprising *TcADK4*, ISs (solid bold lines) and flanking coding sequences (light grey boxes). II, TGRVs produced by replacement of a fragment of *TcADK4* with the SMs of p*Tc*R-HG Hyg - and p*Tc*R-GA Neo -. For clarity equivalent sequences in both constructs are only shown for pL4-GA Neo. III, The TGRVs were linearized and independently transfected into epimastigote cells (arrow). The homologous recombination events between the constructs and the genomic locus are represented with crossed dotted lines. IV, resulting recombinant loci and expected amplification products. Primers 4cF and 4cR bind at positions 382 and 440 in the upstream and downstream genes respectively. Similarly HR, gF, gR and aF are primers specific for the HX1, gapdh II, gapdh I-II and actin ISs. The systematic ID of the flanking genes is given below. V, EEV generated by insertion of the SM of p*Tc*R-HG Neo - upstream the cloned fragment containing *TcADK4*. VI, Tagged EEV obtained after in-frame insertion of eGFP in replacement of a fragment of *TcADK4*. The numbers are the lengths, in base pairs, of the respective element above, the amplification products (in I and IV) or targeting sequences (in III). The letters represent the restriction enzyme sites used in the construction steps. N, NsiI; S, SalI; B, BamHI; C, Csp45I. (B) PCR analysis of genomic DNA from transgenic (T) and wild type (W) epimastigotes. The constructs and primer pairs employed in each case are indicated as well as the relevant lengths of the molecular weight marker bands. (C) Western blot analysis of extracts from wild type (W) and transgenic parasites obtained with pL4x-HG Neo (L4), pL4x-GFP HG Neo (L4G) and pTEXL-Neo (G). Transgenic parasites were cultured with 100 µg/mL of G418. Top panels derive from membranes stained with anti-*Tc*ADK4 (left) or anti-GFP (right) while bottom panels are equivalents stained with anti-PAR2. Below the lanes, *Tc*ADK4 or GFP expression levels relative to those of PAR2 are indicated. Molecular weights are shown.

The p*Tc*R vectors are particularly well suited for the generation of TGRVs. The only additional molecules required are genomic DNA fragments harbouring the genes of interest and optional standard cloning vectors (Vector information S1). To illustrate a simple example of this methodology, TGRVs were produced for the adenylate kinase 4 gene (*TcADK4*) [24]. A ^≈^2.7 Kbp DNA segment containing the gene for *Tc*ADK4 along with ISs and fragments of upstream and downstream coding sequences was cloned into pGEM-T Easy (Promega) (Figure 3A I). Inserting p*Tc*R derived Neo and Hyg SMs in replacement of a section of *TcADK4*, two TGRVs with ^≈^1 Kbp of flanking targeting sequences were obtained (Figure 3A II). These were linearized at restriction enzyme sites of the cloning vector and transfected into epimastigote cells (Figure 3A III and IV). Expected PCR-amplification products corresponding to recombinant regions could be obtained from DNA samples derived from the respective selected cell lines (Figure 3B).

On the other hand p*Tc*R vectors can be easily used for the production of EEVs (Vector Information S1). Inserting a HG Neo SM upstream from the already cloned *TzADK4* genomic fragment the pL4X-HG Neo EEV was obtained (Fig. 3A V). The *Tz*ADK4 expression levels in derived transgenic parasites (Figure 3C, lane L4) were 54 % higher than those in equivalents transfected with pTEXL-Neo (Figure 3C, lane G) produced from the endogenous genes.

### A source of traceable elements: pDIY vectors

The second objective of this work was the development of additional molecular building blocks consisting of traceable elements. Most of the available vectors used for the expression of fusion proteins contain the coding sequence for a single traceable element and, in general, exclusively allow the addition to either the amino- or carboxyl-terminus of the subject protein. Consequently for each fusion protein to be assayed an equal number of vectors has to be acquired or constructed. Additionally, the subject coding sequence has to be specifically made compatible for the adequate incorporation into each different destination vector.

**Figure 4:**
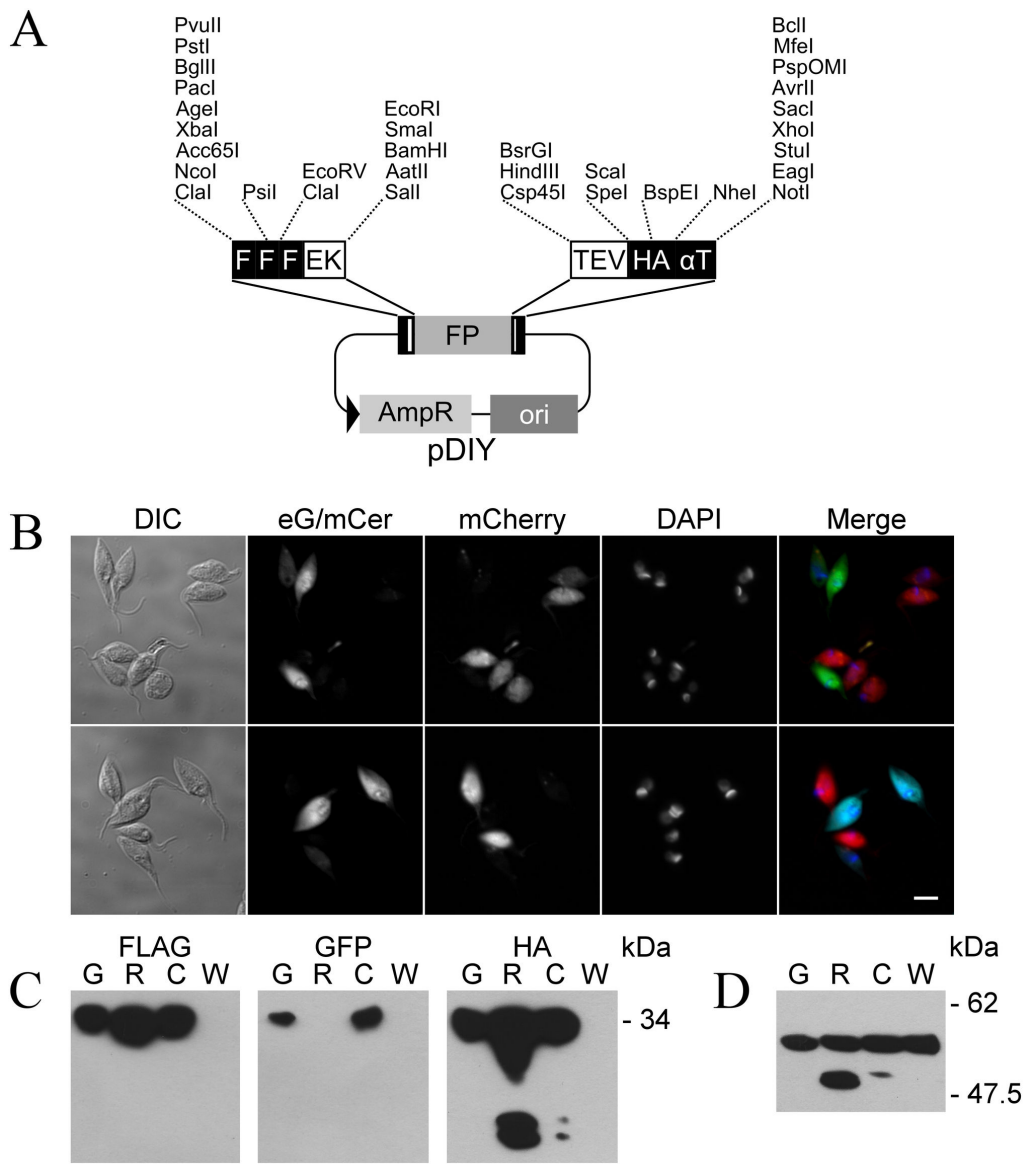
pDIY vectors. (A) Schematic map: The coding sequence contained in the pDIY vectors is represented above. Black boxes represent FLAG (F), the human influenza hemagglutinin (HA) and alpha tubulin (αT) epitopes. White boxes are enterokinase (EK) and tobacco etch virus protease (TEV) cleavage sites. The grey box FP represents any of the three fluorescent protein (eGFP, mCherry and mCerulean) coding sequences available. The minimum backbone vector is schematized with the ampicillin resistance gene (AmpR) and the bacterial replication origin (ori). Useful restriction enzyme sites are indicated. An extended restriction map can be found in Vector Information S1. (B) Microphotographs of epimastigotes expressing fusion proteins involving eGFP (top) and mCerulean (bottom) mixed with equivalents expressing the mCherry variant. The bar at the lower right represents 5 µm. (C) Western blot analysis of whole cell extracts from epimastigotes transgenic for the different fusion proteins. Lanes labelled G (green), R (red) and C (cyan) correspond to parasites expressing eGFP, mCherry and mCerulean respectively. Additionally lane WT indicates samples from the parental epimastigote strain. After electroblotting membranes were stained with anti-FLAG clone M2 mouse monoclonal antibody (FLAG), anti-GFP rabbit polyclonal antibody (GFP) or anti-HA clone 3F10 rat monoclonal antibody (HA). The predicted molecular weight for the fusion protein is indicated. (D) Extracts from transgenic and WT cell analysed with anti-alfa tubulin YL1/2 rat monoclonal antibody. Molecular weight markers closest to the bands are indicated. Microphotographs and extracts were derived from transgenic epimastigotes of the MJ-Levin strain.

The pDIY vectors (Figure 4A) were constructed in order to maximise the number of cloning possibilities and therefore the number of potential resulting fusion proteins with fewer construction reformulation strategies. These molecules can be considered as minimum plasmids containing coding sequences for different traceable elements strategically inserted in frame between the different restriction enzyme sites of an extended MCS. Each pDIY vector contains the coding sequence for a different fluorescent protein and those currently included correspond to the widely used enhanced green eGFP (Clontech) and the strictly monomeric mCherry [25] (Clontech) and mCerulean [26], respective red and cyan newer generation variants with improved brightness and photo-stability. These fluorescent proteins can either be fused to the amino- or carboxy-terminus of proteins of interest. Since the pDIY vectors are identical beyond these coding regions, any of the fluorescent protein genes can be employed in the construction of differently coloured fusion protein expression vectors with essentially the same cloning strategies and, if later required; exchanging these elements in general represents a straight forward task. On each side of the fluorescent protein genes the pDIY vectors contain coding sequences for epitope tags. Respectively at the 5´ and 3´ ends the sequences for the 3-FLAG (Sigma) and influenza virus hemagglutinin HA [27] epitopes were inserted. The entire coding sequence ends with the sequence for the strictly C-terminal alpha tubulin (αT) epitope [28]. For each of the included antigenic determinants immunoaffinity matrices are commercially available and it has been shown that bound tagged proteins or complexes to these can be specifically eluted under gentle nondenaturing conditions by competition with the respective synthetic epitope peptides (3-FLAG [29], FLAG [30], HA [31], αT [32]). Since these elements are all grouped in the same plasmid, the pDIY vectors constitute good starting points for the development not only of single step but multiple sequential step immunoaffinity purification procedures. Therefore considering additional elution alternatives the sequences for the enterokinase (EK) [33] and tobacco etch virus (TEV) [34] protease cleavage sites were placed linking the different traceable elements. 

The pDIY vectors were designed to allow extensive modifications of the harboured coding sequences. Employing standard genetic engineering techniques like digestions with restriction enzymes, optional treatment with DNA polymerases and ligation, segments can be removed or in frame stop codons can be generated. Thus from the original coding sequences at least 23 derived ones can be obtained with different combinations of elements. On the other hand, being capable of admitting DNA fragments with cohesive ends produced by 41 different restriction enzymes, these vectors constitute good genetic construction platforms. The insertion of appropriate regulatory elements and SMs can promote these silent plasmids into expression vectors for different organisms. Alternatively, the coding sequence can be transferred to already available expression systems. In the case of *T. cruzi*, the inducible pTcINDEX [23] and the Gateway (Invitrogen) based p*Tc*GW [11], are suitable recipients. Furthermore the latter can even be extended for C-terminal fusions (see Vector Information S1 for suggested cloning strategies).

To evaluate the performance of the different traceable elements simultaneously expressed as a single fusion protein in *Trypanosoma cruzi*, the coding sequences contained in the three pDIY variants were transferred to pTREX [19] expression vectors. Transgenic epimastigotes of the MJ-Levin were obtained with the resulting plasmids and these showed fluorescence distributed throughout the cell body (Figure 4B). The standard set of filter cubes employed was capable of distinguishing mCherry from the other two fluorescent proteins (both *Aequorea victoria* GFP derivatives). The presence of each epitope tag was assayed by Western-Blot on whole cell extracts (Figure 4C). A band of the predicted molecular weight of the fusion proteins could be detected in membranes respectively stained with anti-FLAG, anti-GFP and anti-HA reactive antibodies. The expression levels reached by the mCherry fusion protein were higher than those observed for the equivalents with eGFP and mCerulean. In these samples additional bands corresponding probably to the proteolytic degradation of mCherry were detected with the anti-HA antibody. Finally when staining the membranes with YL1/2 (Figure 4D), the band corresponding to the tyrosylated form of alpha-tubulin could be identified in extracts from transgenic as well as WT parasites. Under these conditions the expected additional band corresponding to the fusion protein with the αT epitope could not be detected. 

Having so many cloning capabilities it is likely that a gene of interest already contains restriction enzyme sites suitable for the in frame insertion of a pDIY derived traceable element (Vector Information S1). In this way a BamHI site in *TcADK4* allowed the insertion of the eGFP gene of pDIY-eG into pL4X-HG Neo. The eGFP EEV produced was termed pL4X-GFP HG Neo (Figure 3A VI). After the selection period, microscopic examination of epimastigotes transfected with this plasmid revealed fluorescent protein evenly distributed throughout the cell body (data not shown). The intensity of the signal was much lower than that observed in parasites obtained with pTEXL-Neo. Western-Blot assays confirmed lower expression levels as well as the expected increase in molecular weight related to remnants of the N-terminal region of *TcADK4* in the fusion protein (Figure 3C, lanes L4G and G).

### Concluding remarks

The plasmids developed in this work represent an integral contribution to the efforts invested in the genetic manipulation of *Trypanosoma cruzi*. By providing means to setup practical transfection and selection strategies we attempted to help extend the number of strains on which to conduct these methodologies. On the other hand, not seeking to replace but to complement, extend and alternatively work in concert with already available genetic manipulation tools, we developed simple molecular building blocks. Additionally, to encourage creativity, we provided examples illustrating novel ways in which these functional genetic units can be used with sophisticated cloning technologies or simply exploiting the presence of restriction enzyme sites according to cost efficient methodologies. All vectors are available upon request. 

## Supporting Information

Methods S1 **Detailed plasmid construction methods.** A list of all oligonucleotides employed in this work along with the construction steps required for the construction of pTREXL, pTEXL, pMCS, p*Tc*R and pDIY vector series is provided. Corresponding GenBank accession numbers are indicated for each vector.
(PDF)

Results S1 **Transgenic epimastigote microphotographs.** Figures of fluorescent epimastigotes transfected with pTREXL and pTEXL vectors. An example of parasites simultaneously expressing GFP and mCherry fusion proteins from different pTEXL vectors is provided.
(PDF)

Vector Information S1 **Extended restriction maps and general application guidelines.** Detailed maps including restriction enzyme sites present in the MCSs of pTREXL, pTEXL, pT*c*R and pDIY are provided. For each vector, cloning strategies for the development of novel genetic manipulation tools or for the extension of already available genetic constructs are suggested.
(PDF)

## References

[1] Rassi A Jr, Rassi A, Marin-Neto JA (2010) Chagas disease. Lancet 375: 1388-1402. doi:10.1016/S0140-6736(10)60061-X. PubMed: 20399979. 20399979

[2] De Souza W (2002) Basic cell biology of Trypanosoma cruzi. Curr Pharm Des 8: 269-285. doi:10.2174/1381612023396276. PubMed: 11860366. 11860366

[3] Sturm NR, Campbell DA (2010) Alternative lifestyles: the population structure of Trypanosoma cruzi. Acta Trop 115: 35-43. doi:10.1016/j.actatropica.2009.08.018. PubMed: 19695212. 19695212

[4] El-Sayed NM, Myler PJ, Bartholomeu DC, Nilsson D, Aggarwal G et al. (2005) The genome sequence of Trypanosoma cruzi, etiologic agent of Chagas disease. Science 309: 409-415. doi:10.1126/science.1112631. PubMed: 16020725. 16020725

[5] Liang XH, Haritan A, Uliel S, Michaeli S (2003) Trans and cis splicing in trypanosomatids: mechanism, factors, and regulation. Eukaryot Cell 2: 830-840. 14555465 10.1128/EC.2.5.830-840.2003 PMC219355

[6] Clayton CE (2002) Life without transcriptional control? From fly to man and back again. EMBO J 21: 1881-1888. doi:10.1093/emboj/21.8.1881. PubMed: 11953307. 11953307 PMC125970

[7] Kelly JM, Ward HM, Miles MA, Kendall G (1992) A shuttle vector which facilitates the expression of transfected genes in Trypanosoma cruzi and Leishmania. Nucleic Acids Res 20: 3963-3969. doi:10.1093/nar/20.15.3963. PubMed: 1324472. 1324472 PMC334073

[8] Martínez-Calvillo S, López I, Hernández R (1997) pRIBOTEX expression vector: a pTEX derivative for a rapid selection of Trypanosoma cruzi transfectants. Gene 199: 71-76. doi:10.1016/S0378-1119(97)00348-X. PubMed: 9358041. 9358041

[9] Lorenzi HA, Vazquez MP, Levin MJ (2003) Integration of expression vectors into the ribosomal locus of Trypanosoma cruzi. Gene 310: 91-99. doi:10.1016/S0378-1119(03)00502-X. PubMed: 12801636. 12801636

[10] Xu D, Brandán CP, Basombrío MA, Tarleton RL (2009) Evaluation of high efficiency gene knockout strategies for Trypanosoma cruzi. BMC Microbiol 9: 90. doi:10.1186/1471-2180-9-90. PubMed: 19432966. 19432966 PMC2688506

[11] Batista M, Marchini FK, Celedon PA, Fragoso SP, Probst CM et al. (2010) A high-throughput cloning system for reverse genetics in Trypanosoma cruzi. BMC Microbiol 10: 259. doi:10.1186/1471-2180-10-259. PubMed: 20942965. 20942965 PMC3020659

[12] DaRocha WD, Otsu K, Teixeira SM, Donelson JE (2004) Tests of cytoplasmic RNA interference (RNAi) and construction of a tetracycline-inducible T7 promoter system in Trypanosoma cruzi. Mol Biochem Parasitol 133: 175-186. doi:10.1016/j.molbiopara.2003.10.005. PubMed: 14698430. 14698430

[13] Camargo EP (1964) Growth and Differentiation in Trypanosoma Cruzi. I. Origin of Metacyclic Trypanosomes in Liquid Media. Rev Inst Med Trop Sao Paulo 6: 93-100. PubMed: 14177814. 14177814

[14] Lennox ES (1955) Transduction of linked genetic characters of the host by bacteriophage P1. Virology 1: 190-206. doi:10.1016/0042-6822(55)90016-7. PubMed: 13267987. 13267987

[15] Birnboim HC, Doly J (1979) A rapid alkaline extraction procedure for screening recombinant plasmid DNA. Nucleic Acids Res 7: 1513-1523. doi:10.1093/nar/7.6.1513. PubMed: 388356. 388356 PMC342324

[16] Laemmli UK (1970) Cleavage of structural proteins during the assembly of the head of bacteriophage T4. Nature 227: 680-685. doi:10.1038/227680a0. PubMed: 5432063. 5432063

[17] Rice P, Longden I, Bleasby A (2000) EMBOSS: the European Molecular Biology Open Software Suite. Trends Genet 16: 276-277. doi:10.1016/S0168-9525(00)02024-2. PubMed: 10827456. 10827456

[18] Aslett M, Aurrecoechea C, Berriman M, Brestelli J, Brunk BP et al. (2010) TriTrypDB: a functional genomic resource for the Trypanosomatidae. Nucleic Acids Res 38: D457-D462. doi:10.1093/nar/gkp851. PubMed: 19843604. 19843604 PMC2808979

[19] Vazquez MP, Levin MJ (1999) Functional analysis of the intergenic regions of TcP2beta gene loci allowed the construction of an improved Trypanosoma cruzi expression vector. Gene 239: 217-225. doi:10.1016/S0378-1119(99)00386-8. PubMed: 10548722. 10548722

[20] Cevallos AM, López-Villaseñor I, Espinosa N, Herrera J, Hernández R (2003) Trypanosoma cruzi: allelic comparisons of the actin genes and analysis of their transcripts. Exp Parasitol 103: 27-34. doi:10.1016/S0014-4894(03)00066-3. PubMed: 12810043. 12810043

[21] Cevallos AM, Segura-Kato YX, Merchant-Larios H, Manning-Cela R, Alberto Hernandez-Osorio L et al. (2011) Trypanosoma cruzi: multiple actin isovariants are observed along different developmental stages. Exp Parasitol 127: 249-259. doi:10.1016/j.exppara.2010.08.003. PubMed: 20705070. 20705070

[22] Hamilton MD, Nuara AA, Gammon DB, Buller RM, Evans DH (2007) Duplex strand joining reactions catalyzed by vaccinia virus DNA polymerase. Nucleic Acids Res 35: 143-151. doi:10.1093/nar/gkm378. PubMed: 17158165. 17158165 PMC1802553

[23] Taylor MC, Kelly JM (2006) pTcINDEX: a stable tetracycline-regulated expression vector for Trypanosoma cruzi. BMC Biotechnol 6: 32. doi:10.1186/1472-6750-6-32. PubMed: 16824206. 16824206 PMC1544328

[24] Bouvier LA, Miranda MR, Canepa GE, Alves MJ, Pereira CA (2006) An expanded adenylate kinase gene family in the protozoan parasite Trypanosoma cruzi. Biochim Biophys Acta 1760: 913-921. doi:10.1016/j.bbagen.2006.02.013. PubMed: 16567051. 16567051

[25] Shaner NC, Campbell RE, Steinbach PA, Giepmans BN, Palmer AE et al. (2004) Improved monomeric red, orange and yellow fluorescent proteins derived from Discosoma sp. red fluorescent protein. Nat Biotechnol 22: 1567-1572. doi:10.1038/nbt1037. PubMed: 15558047. 15558047

[26] Rizzo MA, Springer GH, Granada B, Piston DW (2004) An improved cyan fluorescent protein variant useful for FRET. Nat Biotechnol 22: 445-449. doi:10.1038/nbt945. PubMed: 14990965. 14990965

[27] Wilson IA, Niman HL, Houghten RA, Cherenson AR, Connolly ML et al. (1984) The structure of an antigenic determinant in a protein. Cell 37: 767-778. doi:10.1016/0092-8674(84)90412-4. PubMed: 6204768. 6204768

[28] Wehland J, Schröder HC, Weber K (1984) Amino acid sequence requirements in the epitope recognized by the alpha-tubulin-specific rat monoclonal antibody YL 1/2. EMBO J 3: 1295-1300. PubMed: 6204858. 6204858 10.1002/j.1460-2075.1984.tb01965.x PMC557511

[29] Hall MC, Torres MP, Schroeder GK, Borchers CH (2003) Mnd2 and Swm1 are core subunits of the Saccharomyces cerevisiae anaphase-promoting complex. J Biol Chem 278: 16698-16705. doi:10.1074/jbc.M213109200. PubMed: 12609981. 12609981

[30] Einhauer A, Jungbauer A (2001) The FLAG peptide, a versatile fusion tag for the purification of recombinant proteins. J Biochem Biophys Methods 49: 455-465. doi:10.1016/S0165-022X(01)00213-5. PubMed: 11694294. 11694294

[31] Field J, Nikawa J, Broek D, MacDonald B, Rodgers L et al. (1988) Purification of a RAS-responsive adenylyl cyclase complex from Saccharomyces cerevisiae by use of an epitope addition method. Mol Cell Biol 8: 2159-2165. PubMed: 2455217. 2455217 10.1128/mcb.8.5.2159-2165.1988 PMC363397

[32] Stammers DK, Tisdale M, Court S, Parmar V, Bradley C et al. (1991) Rapid purification and characterisation of HIV-1 reverse transcriptase and RNaseH engineered to incorporate a C-terminal tripeptide alpha-tubulin epitope. FEBS Lett 283: 298-302. doi:10.1016/0014-5793(91)80613-8. PubMed: 1710580. 1710580

[33] Light A, Savithri HS, Liepnieks JJ (1980) Specificity of bovine enterokinase toward protein substrates. Anal Biochem 106: 199-206. doi:10.1016/0003-2697(80)90138-4. PubMed: 6998318. 6998318

[34] Parks TD, Howard ED, Wolpert TJ, Arp DJ, Dougherty WG (1995) Expression and purification of a recombinant tobacco etch virus NIa proteinase: biochemical analyses of the full-length and a naturally occurring truncated proteinase form. Virology 210: 194-201. doi:10.1006/viro.1995.1331. PubMed: 7793070. 7793070

[35] Nozaki T, Cross GA (1994) Functional complementation of glycoprotein 72 in a Trypanosoma cruzi glycoprotein 72 null mutant. Mol Biochem Parasitol 67: 91-102. doi:10.1016/0166-6851(94)90099-X. PubMed: 7838187. 7838187

[36] Caler EV, Vaena de Avalos S, Haynes PA, Andrews NW, Burleigh BA (1998) Oligopeptidase B-dependent signaling mediates host cell invasion by Trypanosoma cruzi. EMBO J 17: 4975-4986. doi:10.1093/emboj/17.17.4975. PubMed: 9724634. 9724634 PMC1170826

[37] Wilkinson SR, Taylor MC, Horn D, Kelly JM, Cheeseman I (2008) A mechanism for cross-resistance to nifurtimox and benznidazole in trypanosomes. Proc Natl Acad Sci U S A 105: 5022-5027. doi:10.1073/pnas.0711014105. PubMed: 18367671. 18367671 PMC2278226

