# Supplementary material for: Plasmid Vectors and Molecular Building Blocks for the Development of Genetic Manipulation Tools for Trypanosoma cruzi
Source: PLoS One. 2013 Oct 24;8(10):e80217. doi: 10.1371/journal.pone.0080217 (PMC3812015; doi:10.1371/journal.pone.0080217)
Supplement: Methods S1 — Detailed plasmid construction methods. A list of all oligonucleotides employed in this work along with the construction steps required for the construction of pTREXL, pTEXL, pMCS, pTcR and pDIY vector series is provided. Corresponding GenBank accession numbers are indicated for each vector. (PDF) [file pone.0080217.s001.pdf]

Additional materials and methods

Oligonucleotides employed as PCR primers

Name	Sequence 5´ → 3´	Tm	Restriction sites
**4F**	GCTACTCACGAGGCGGTGTT	54.3	---
**4R**	CACTCTGCGTCTATCGTTGTACC	53.2	---
**4cF**	TATTGTTGCGGGTGGTGTTG	54.1	---
**4cR**	ATCTTGCGCTCTTGCCACTG	55.4	---
**HR**	TCTAGACATCTGACAAGACAACTTATAGAGC	55.7	XbaI
**gF**	TTTCCTAGGCGTGGCGATGACTTC	62.2	AvrII
**gR**	CGCTGTGTCAgTTGGATCGTG	56.3	d-MfeI
**IG1G2T2CF**	CACGATCCAAcTGACACAGCG	56.3	d-MfeI
**IG1G2R**	ATGCATTTTAAATTCTGTTCAATGTAATTG	56.6	NsiI
**BSF**	TGAACAGAATTTAAAATGGCCAAGCCTTTGTCT	66.4	DraI
**RBR**	TTTGCTAGCCCTCCCACACATAACC	61.1	NheI
**PSF**	TGAACAGAATTTAAAATGGCCAAGTTGACCAGTG	67.3	DraI
**RPR**	TTTACTAGTCCTGCTCCTCGGCC	57.7	SpeI
**HSF**	TGAACAGAATTTAAAATGAAAAAGCCTGAACTCACC	66.1	DraI
**RHR**	TTTACTAGTCCTTTGCCCTCGGACG	60.6	SpeI
**NSF**	TGAACAGAATTTAAAATGCTTGAACAAGATGG	62.4	DraI
**RNR**	TTTTCTAGAAGAACTCGTCAAGAAGGC	57.0	XbaI
**RSF**	TGAACAGAATTTAAAATGACCGAGTACAAGCC	63.2	DraI
**RRR**	TTTCCTAGGCACCGGGCTTGCG	65.7	AvrII
**GHF**	AAGCTTAGGCGTGGCGATGACTTC	61.6	HindIII
**IG2R**	GCTAGCTTGCATGCATAATGCACAAACACTAAACTAC	67.0	NheI-NsiI
**BBF**	TTTGCGGCCGCTCTTCCGCTTCCTCG	74.4	NotI
**BBR**	TTTGCGGCCGCGGAACCCCTATTTGTTTA	72.3	NotI
**HX1HF**	AAGCTTAACGAGTTTCTTCAAAATAT	51.2	HindIII
**G2HR**	AAGCTTAATGCACAAACACTAAACT	51.0	HindIII
**aF**	TTTACTAGTAATCTCTACATAAAGGTGA	47.5	SpeI
**IA1A2R**	ATGCATCCTTAGAAACAAAAAAAA	51.5	NsiI
**AHR**	AAGCTTAGAAACAAAAAAAAAAAAAAC	52.5	HindIII
**eGFPF**	GTCGACGTGAGCAAGGGCGAGGAG	66.7	SalI
**eGFPR**	AAGCTTTTGTACAGCTCGTCCATGCC	62.5	HindIII
**XATGS-eGFPF**	TTTTCTAGATGGTCGACGTGAGCAAGGGC	67.4	XbaI-SalI
**3FKNF**	GGTACCATGGGCATCGATTACAAAGACC	64.0	KpnI
**BBPPR**	CTGCAGCTGAACCCCTATTTGTTTATTTTTC	62.9	PstI
**mChYF**	GTCGACAAAGGAGAAGAAAATAACgTGGCAA	65.6	SalI
**mChYR**	AAGCTTTTGTACAGTTCATCCATGCCACC	63.9	HindIII
**S72AF**	GCAGTGCTTCgcCCGCTACCCC	67.3	---
**S72AR**	GGGGTAGCGGgcGAAGCACTGC	67.3	---

**Y145A+H148DF**	GGAGTACAACgcCATCAGCgACAACGTCTA	68.0	---
**Y145A+H148DR**	TAGACGTTGTcGCTGATGgcGTTGTACTCC	68.0	---
**A206KF**	CACCCAGTCCaagCTGAGCAAAG	59.8	---
**A206KR**	CTTTGCTCAGcttGGACTGGGTG	59.8	---

For each oligonucleotide the predicted Tm and restriction enzyme site used in the constructions steps are indicated on the right. The sites are additionally shown underlined in the sequences, except for gR and IG1G2T2CF where the MfeI site to be removed is highlighted instead. Mutagenic nucleotides are indicated with lowercase characters.

Oligonucleotides employed as linkers

Name	Sequence 5´ → 3´	Tm	Restriction sites	
			5´	3´
**MCS1**	GGCCGCAGCTGGATCCATGGTACCGATATCGC	76.4	NotI	NotI
	GGCCGCGATATCGGTACCATGGATCCAGCTGC	76.4		
**MCS2a**	GATCCTCGAGCTAGCAATTGTACATGCAT	62.5	BamHI	ClaI
	CGATGCATGTACAATTGCTAGCTCGAG	61.9		
**MCS2b**	CGATCTAGATCTGCAGTCGACCATGGTAC	62.9	ClaI	KpnI
	CATGGTCGACTGCAGATCTAGAT	52.6		
**MCS3**	TCGATCATGAAGCTTCGAATT	51.6	NsiI	XbaI
	CTAGAATTCGAAGCTTCATGATCGATGCA	63.4		
**HA**	AGCTTAATCACTAGTTACCCCTACGACGTTCCGGATTACGCTAGCTGAC	75.7	HindIII	XhoI
	TCGAGTCAGCTAGCGTAATCCGGAACGTCGTAGGGGTAACTAGTGATTA	76.9		
**aT**	CTAGCGGCGAAGAGTTCTGATCAATTGATCCGAGC	72.7	NheI	XhoI
	TCGAGCTCGGATCAATTGATCAGAACTCTTCGCCG	75.0		
**3-FLAG-N**	CTAGATGGGCATCGATTACAAAGACCATGACGGTGATTATAAA	72.1	XbaI	BglII
	GATCTTTATAATCACCGTCATGGTCTTTGTAATCGATGCCCAT	72.6		
**3-FLAG-C**	GATCATGATATCGATTACAAGGATGACGATGACAAGG	68.2	BclI	EcoRI
	AATTCCTTGTCATCGTCATCCTTGTAATCGATATCAT	66.8		
**MCS**	AATTCCCGGGATCCGACG	56.7	EcoRI	SalI
	TCGACGTCGGATCCCGGG	61.1		
**TEV**	AGCTTCGAAAATCTGTACTTTCAGGGAAGTA	61.1	HindIII	SpeI
	CTAGTACTTCCCTGAAAGTACAGATTTTCGA	58.3		
**MCS-Nt**	GATCTTAATTAACCGGTCTAGAGGTAC	52.0	PstI	KpnI
	CTCTAGACCGGTTAATTAAGATCTGCA	55.7		
**MCS-Ct**	AATTGGGCCCTAGGAGCTCGAGGCCTGC	71.9	MfeI	NotI
	GGCCGCAGGCCTCGAGCTCCTAGGGCCC	77.7		

For each linker the sequence of the constituting complementary oligonucleotide pair is indicated as well as the respective predicted Tm. Once annealed the fragments contain

cohesive ends equivalents to those obtained by restriction with the enzymes shown on the right.

Linker preparation

Corresponding oligonucleotides were prepared as 100 µM stock solutions and 1 µl of each were incubated overnight at 37 ºC with 5 U of PNK in a final volume of 10 µl ATP containing 1X T4 DNA ligase buffer. Both samples were mixed and suplemented with 2.2 µl of 10X annealing buffer (100 mM Tris-HCl, pH 8; 10 mM EDTA, 2 M NaCl). To anneal the oligonucleotides, in a thermal cycler, the mixture was heated at 95 ºC for 5 minutes and then the temperature was made to descend 1 ºC/min until 4 ºC were reached. Afterwards the samples were stored at -20 ºC. For each ligation the linkers were diluted 1/25 in 1X annealing buffer and 2.2 µl of the dilution were used in 10 µl ligation reactions involving between 20 ng and 50 ng of linearized vectors.

Plasmid construction

Given the large number of cloning steps standard DNA manipulation methods were omitted. Agarose gel DNA fragment purification was performed with GenElute Gel Extraction Kit (Sigma). Most PCR amplifications were done with Pfu DNA polymerase and consequently prior to inserting into the T/A pGEM-T Easy cloning vector the purified fragments were incubated for 1 h at 72 ºC with Taq DNA polymerase. After certain key steps the constructs were sent to Macrogen for sequencing.

Construction of pTREXL vectors

A *T. cruzi* fluorescent protein expression vector was obtained by inserting the 763 bp *XbaI* fragment containing the eGFP gene from pEGFP (Clontech) into the *XbaI* site of pTREX (kindly provided by Dr. M. Vazquez). A clone with the gene in the sense orientation was designated pTREX-eGFP. To remove remnants of coding sequences and an *MfeI* site contained in the gapdh I-II IS of pTREX, two fragments (117 bp and 432 bp long) were PCR-amplified with the respective primer pairs IG1G2F-gR and IG1G2T2CF- IG1G2R using pTREX-eGFP as template. Both products were combined and fused in an overlap extension PCR reaction involving primers gF and IG1G2R. The 514 bp product was cloned into pGEM-T Easy (Promega) and this plasmid was designated Teasy-gapdh I-II. The IS was reamplified with primers GHF and IG1G2R. On the other hand, the BSD, ble, Hyg, Neo and PAC resistance genes were isolated from (respectively) plasmids pUB/BSD (Invitrogen), pZErO-1 (Invitrogen), pSilencer 2.1-U6 hygro (Ambion), pREP4 (QIAGEN), and pTREX-PAC (kindly provided by Dr. H. Lorenzi) by PCR with primer pairs BSF-RBR, PSF-RPR, HSF-RHR, NSF-RNR and RSF-RRR. These amplification products were spliced by overlap extension to the previously reampliflied I gapdh I-II region in PCR reactions involving oligonucleotide GHF as forward primer and the respective RBR, RPR, RHR, RNR and RRR oligonucleotides as reverse primers. The resulting fragments were cloned into pGEM-T Easy and consisted in the resistance genes with the gapdh I-II IS at their 5´ ends. From each ligation, a clone with the insert in the orientation of the T7

promoter transcription was recovered and named respectively Teasy-G-BSD, Teasy-G-ble, Teasy-G-Hyg, Teasy-G-Neo and Teasy-G-PAC.

Similarly, the IS gapdh II was amplified from pTREX-eGFP with primers gF and IG2R and later on, cloned into pGEM-T Easy. A clone with the insert in opposite orientation relative to the transcription of the T7 promoter was recovered and designated Teasy-gapdh II. The gapdh II region in this plasmid was transferred as a NheI-PstI fragment to pTREX-eGFP previously digested with the same enzymes. The resulting vector was named pTREX-eGFP-GII. On the other hand, a similar fragment obtained by digestion of Teasy-gapdh II with NsiI and AvrII was inserted into Teasy-G-BSD, Teasy-G-ble, Teasy-G-Hyg, Teasy-G-Neo and Teasy-G-PAC. These had previously been digested with NsiI in combination with NheI (BSD), SpeI (ble and Hyg), XbaI (Neo) or AvrII (PAC). The obtained plasmids were named Teasy-G-BSD-G, Teasy-G-ble-G, Teasy-G-Hyg-G, Teasy-G-Neo-G and Teasy-G-PAC-G. Finally the SMs composed of the resistance genes with gapdh I-II and gapdh II ISs at their 5´ and 3´ ends were transferred as HindIII-NsiI segments from these Teasy plasmids to pTREX-eGFP-GII previously digested with the same enzymes. The resulting vectors were designated pTREXL-BSD, pTREXL-ble, pTREXL-Hyg, pTREXL-Neo and pTREXL-PAC.

Accession numbers	
pTREXL-BSD	JN596092
pTREXL-ble	JN596095
pTREXL-Hyg	JN596093
pTREXL-Neo	JN596094
pTREXL-PAC	JN596096

Construction of pTEXL vectors

A 458 bp SacI-BamHI fragment containing the gapdh I IS of pTEX was transferred from pTEX-TAP (kindly provided by Dr. E. Serra) to pTREXL-BSD previously digested with the same enzymes in replacement of the 1033 bp region comprising the ribosomal promoter and HX1 sequence. This vector was called pTEXL-BSD. On the other hand the fluorescent protein gene and downstream SMs of pTREXL-ble, pTREXL-Hyg, pTREXL-Neo and pTREXL-PAC were produced as BamHI-NsiI segments (with length respectively of 2188 bp, 2839 bp, 2608 bp, and 2413 bp) and inserted into the backbone vector obtained by digestion of pTEXL-BSD with the same enzymes. The resulting vectors were designated respectively pTEXL-ble, pTEXL-Hyg, pTEXL-Neo and pTEXL-PAC.

Accession numbers	
pTEXL-BSD	JN596097
pTEXL-ble	JN596100
pTEXL-Hyg	JN596098
pTEXL-Neo	JN596099
pTEXL-PAC	JN596101

Construction of p*Tc*R vectors

pMCS

Using the pUC19 vector as template a 1928 bp fragment containing the ampicillin SM and bacterial replication origin was PCR-amplified with primers BBF and BBR. After digestion with NotI the molecule was religated and used to transform *E. coli* DH5α bacteria. The obtained plasmid was named pNotI. The MCS1 linker was inserted into this vector, previously digested with NotI, producing pMCS1. Similarly, the linkers MCS2a and MCS2b were inserted simultaneously into BamHI-KpnI digested pMCS1. The product of this three fragment ligation was called pMCS2 and transformed into *E. coli* SCS110 (Stratagene) methylase deficient bacteria. Finally the pMCS vector was obtained with the purified XbaI-NsiI digested pMCS2 by insertion of the MCS3 linker.

p*Tc*R-HG

To remove the HX1 sequence, eGFP gene and gapdh I-II IS each pTREXL vector was digested with XbaI, treated with Klenow DNA polymerase and partially digested with DraI. The resulting 4826 bp, 4802 bp, 5453 bp, 5222 bp and 5027 bp bands obtained respectively from the BSD, ble, Hyg, Neo and PAC variants were purified and blunt religated producing the molecules designated pRPH-BSD-G, pRPH-ble-G, pRPH-Hyg-G, pRPH-Neo-G and pRPH-PAC-G. In PCR reactions involving these molecules as templates and oligonucleotides HX1HF and G2HR as primers the different HG SMs were amplified. These were cloned into pGEM-T Easy producing Teasy-HG-BSD, Teasy-HG-ble, Teasy-HG-Hyg, Teasy-HG-Neo and Teasy-HG-PAC. Finally the HG SMs were transferred as HindIII segments to pMCS previously linearized with the same enzyme. The resulting molecules were named p*Tc*R-HG series. Plasmids with inserts in any of the two possible orientations were recovered.

p*Tc*R-GA

The IS of the *actin 1* and *actin 2* tandem copy genes was PCR-amplified from *T. cruzi* genomic DNA using primers aF and IA1A2R. The 465 bp fragment was cloned into pGEM-T Easy producing Teasy-actin. This plasmid was then digested with SpeI and NsiI and the fragment containing the IS was inserted into Teasy-G-BSD, Teasy-G-ble, Teasy-G-Hyg, Teasy-G-Neo and Teasy-G-PAC which had been digested with NsiI in combination with NheI (BSD), SpeI (ble and Hyg), XbaI (Neo) or AvrII (PAC). The resulting molecules were named Teasy-G-BSD-A, Teasy-G-ble-A, Teasy-G-Hyg-A, Teasy-G-Neo-A and Teasy-G-PAC-A and were employed as template in PCR reactions involving primers GHF and AHR. The amplified GA SMs were cloned into pGEM-T Easy and the resulting molecules were called Teasy-GA-BSD, Teasy-GA-ble, Teasy-GA-Hyg, Teasy-GA-Neo and Teasy-GA-PAC. From each plasmid the SMs were subcloned as HindIII fragments into HindIII digested pMCS producing the p*Tc*R-GA vector series. Plasmids with inserts in any of the two possible orientations were recovered.

Accession numbers				
p*Tc*R	Series			
	HG		GA	
Resistance gene	+	-	+	-
BSD	JN596069	JN596070	JN596079	JN596080
ble	JN596075	JN596076	JN596085	JN596086
Hyg	JN596071	JN596072	JN596081	JN596082
Neo	JN596073	JN596074	JN596083	JN596084
PAC	JN596077	JN596078	JN596087	JN596088

Construction of pDIY vectors

Inserting the HA linker into pTREX digested with HindIII and XhoI, the pTREX-HA vector was obtained. This plasmid was digested with XhoI and partially digested with NheI. The 6241 bp fragment was purified and employed in a ligation involving linker aT. The resulting vector was named pTREX-HA-aT. On the other hand using pTREX-eGFP as template the eGFP gene was amplified with primers eGFPF and eGFPR and the product was ligated into pGEM-T Easy producing Teasy-eGFP. Similarly this vector was included in a PCR reaction with primers XATGS-eGFPF and eGFPR and the resulting fragment was ligated into pGEM-T Easy. A clone with the insert in the orientation of the T7 promoter transcription was recovered and named Teasy-XATGS-eGFP. A section containing the eGFP gene was produced from this plasmid by digestion with EcoRI and HindIII and inserted into pTREX-HA-aT previously digested with the same enzymes. The recovered vector was named pTREX-eGFP-HA-aT. The pTEX-TAP plasmid was linearized with XbaI and BglII and ligated to the 3-FLAG-N linker generating pTEX-3-FLAG-N. A fragment containing coding sequence remnants including those for the partial 3-FLAG epitope and the SM of pTEX was transferred from this vector as a XbaI-Csp45I fragment to pTREX prepared with the same enzymes. From this step pTREX-3-FLAG-N was obtained. This molecule was digested with BglII and EcoRI and later the 3-FLAG-C linker was inserted producing pTREX-3-FLAG. A fragment containing the eGFP-HA-aT coding sequence was prepared by digestion of pTREX-eGFP-HA-aT with EcoRI and XhoI and cloned into pTREX-3-FLAG previously treated with the same enzymes. The recovered molecule was designated pTREX-3-FLAG-eGFP-HA-aT. Further on this plasmid was digested with EcoRI and SalI and the MCS linker was inserted, producing pTREX-3-FLAG-MCS-eGFP-HA-aT. Analogously the TEV linker was cloned into this molecule digested with HindIII and SpeI. For simplicity the resulting molecule (pTREX-3-FLAG-MCS-eGFP-TEV-HA-aT) was named pTREX-Omni-I. With primers 3FKNF and gR the coding sequence and a fragment of gapdh I-II IS was PCR-amplified from pTREX-Omni-I. After ligation to pGEM-T Easy a clone with the insert in the orientation of the T7 promoter transcription was named Teasy-Omni-I. On the other hand pEGFP was digested with PstI and KpnI and the purified fragment was ligated to linker MCS-Nt generating pEGFP-KN. The coding sequence of

Teasy-Omni-I was produced as a KpnI-NotI fragment and inserted into pEGFP-KN, previously digested with the same enzymes, in replacement of the eGFP gene. The thereby obtained pKN-Omni was further digested with MfeI and NotI and the linker MCS-Ct was cloned into it producing pKN-Omni-MN. The bacterial replication origin and ampicillin SM was amplified as 1906 bp segment from pUC19 with primers BBF and BBPPR. In a ligation including PNK the amplification product was recircularized into the minimum plasmid pMNP. A PstI-NotI fragment containing the coding sequence of pKN-Omni-MN was inserted into pMNP which had previously been digested with the same enzymes. The product of this ligation was designated pDIY-eG. A PCR fragment cloning plasmid with the mCherry gene (kindly provided by S. Degese), originally derived from a yeast expression vector was used as template in PCR reactions involving primers mChYF and mChYR. The plasmid obtained after ligating the amplification product into pGEM-T Easy was named Teasy-mCherry. The mCherry gene was transferred to pDIY-eG digested with SalI and HindIII in replacement of the eGFP gene as a fragment derived from Teasy-mCherry treated with the same enzymes. This plasmid was called pDIY-mCh. Using primer pairs eGFPF-S72AR, S72AF- Y145A+H148DR, Y145A+H148DF-A206KR and A206KF-eGFPR in PCR reactions involving pECFP-C1 (Clontech) as template the respective 231 bp, 249 bp, 206 bp and 114 bp amplification products were obtained. The four fragments were combined and fused by overlap extension with primers eGFPF and eGFPR. With this methodology the eCFP gene was mutagenized into the mCerulean derivative. After ligating the amplification product into pGEM-T Easy the Teasy-mCerulean plasmid was obtained. The mCherry gene of pDIY-mCh was removed by digestion with SalI and HindIII and replaced with mCerulean obtained from Teasy-mCerulean treated with the same enzymes. The vector obtained was named pDIY-mCer. An unrelated fragment cloned in pGEM-T Easy was transferred as an XbaI-SalI fragment into pTREX previously digested with XbaI and XhoI. As a result of ligation of the compatible end restriction enzyme sites SalI and XhoI the obtained plasmid lacked sites for the latter enzyme but had acquired the SpeI, EcoRI, NotI and PstI sites from the MCS of pGEM-T Easy. This molecule was digested with XbaI and NotI and the coding sequences of pDIY-eG, pDIY-mCh and pDIY-mCer were inserted into it as fragments generated with the same enzymes. These plasmids were respectively named pTREX-eG, pTREX-mCh and pTREX-mCer.

**Accession numbers**			
	**Fluorescent protein**		
	**eGFP (eG)**	**mCherry (mCh)**	**mCerulean (mCer)**
**pDIY**	JN596089	JN596091	JN596090
