# Supplementary material for: Plasmid Vectors and Molecular Building Blocks for the Development of Genetic Manipulation Tools for Trypanosoma cruzi
Source: PLoS One. 2013 Oct 24;8(10):e80217. doi: 10.1371/journal.pone.0080217 (PMC3812015; doi:10.1371/journal.pone.0080217)
Supplement: Results S1 — Transgenic epimastigote microphotographs. Figures of fluorescent epimastigotes transfected with pTREXL and pTEXL vectors. An example of parasites simultaneously expressing GFP and mCherry fusion proteins from different pTEXL vectors is provided. (PDF) [file pone.0080217.s002.pdf]

Supplementary results:

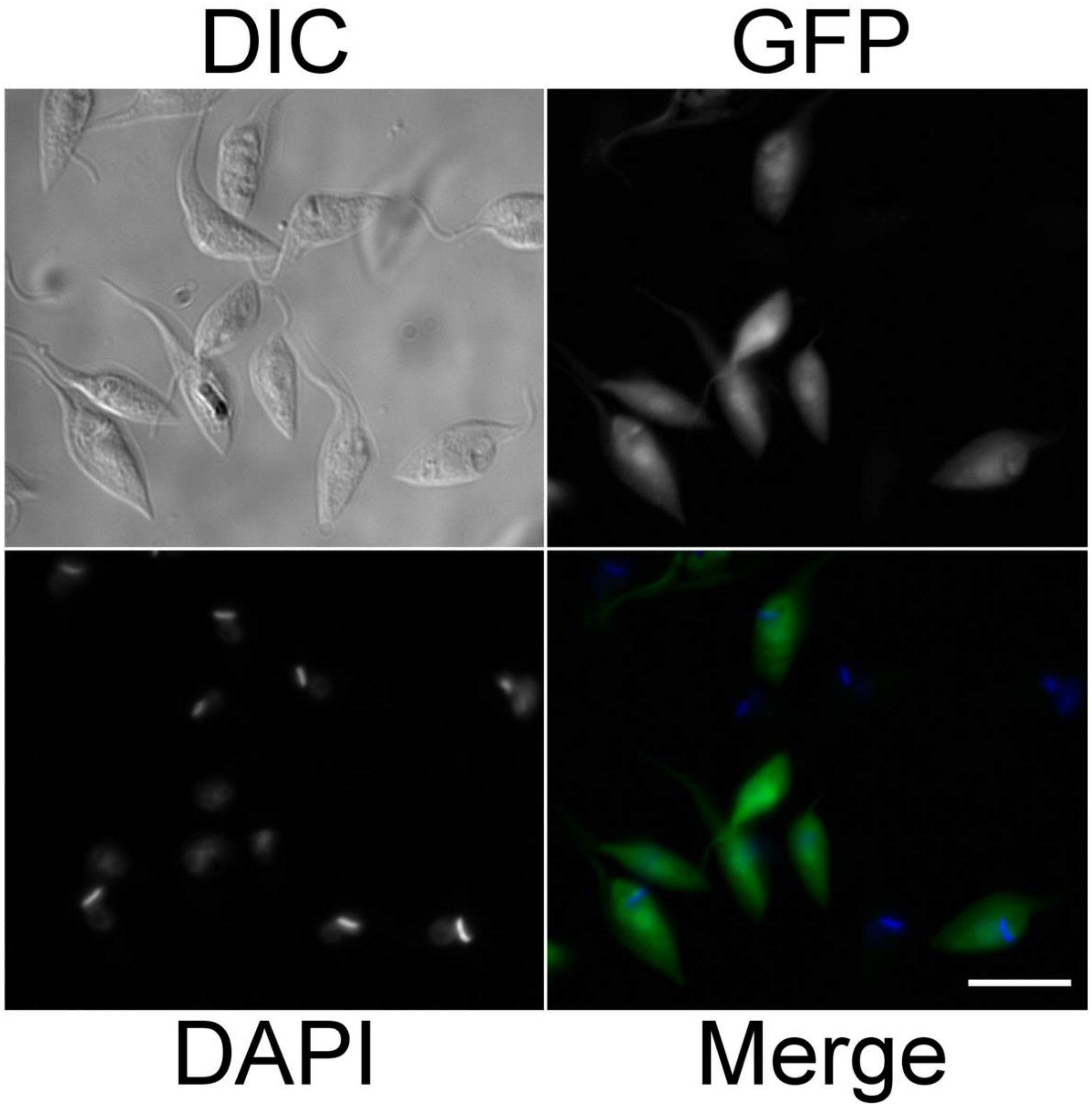

Supplementary Figure R1: Microphotography of MJ-Levin strain epimastigotes transfected with pTREXL-Neo after selection with 500 µg/mL of G418. The bar on the lower right panel corresponds to 10 µm.

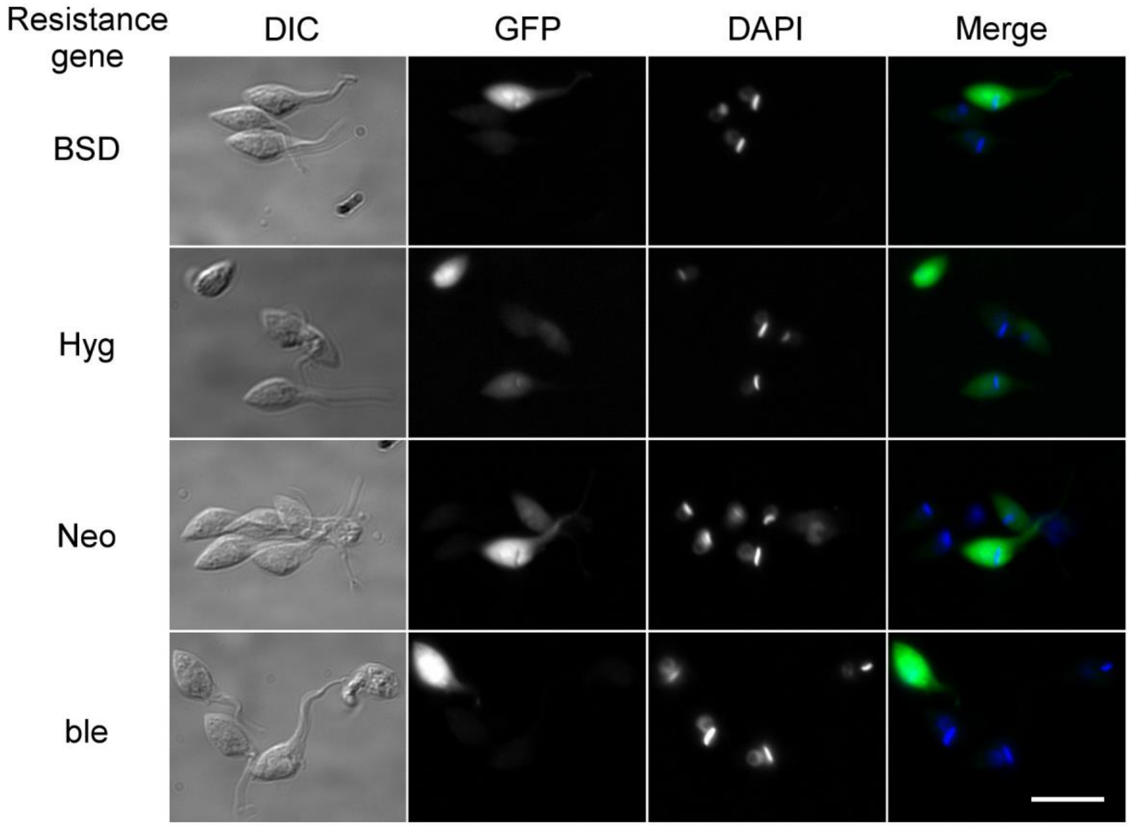

Supplementary Figure R2: Microphotography of MJ-Levin strain epimastigotes transfected with pTEXL-BSD, pTEXL-Hyg, pTEXL-Neo and pTEXL-ble after selection with 50 µg/mL, 500 µg/mL, 200 µg/mL and 250 µg/mL of blasticidin S, hygromycin B, G418 and phleomicin respectively. The bar at the lower right panel corresponds to 10 µm.

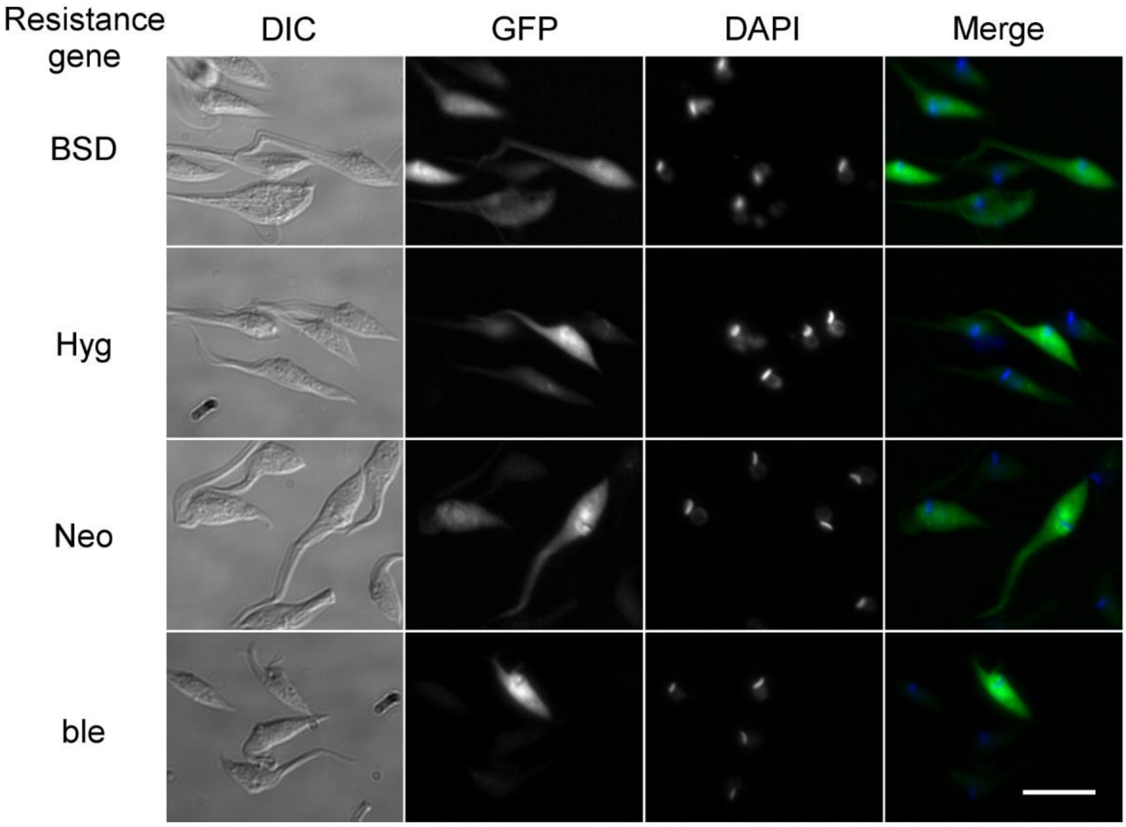

Supplementary Figure R3: Microphotography of Y strain epimastigotes transfected with pTEXL-BSD, pTEXL-Hyg, pTEXL-Neo and pTEXL-ble after selection with 50 µg/mL, 500 µg/mL, 200 µg/mL and 250 µg/mL of blasticidin S, hygromycin B, G418 and phleomicin respectively. The bar at the lower right panel corresponds to 10 µm.

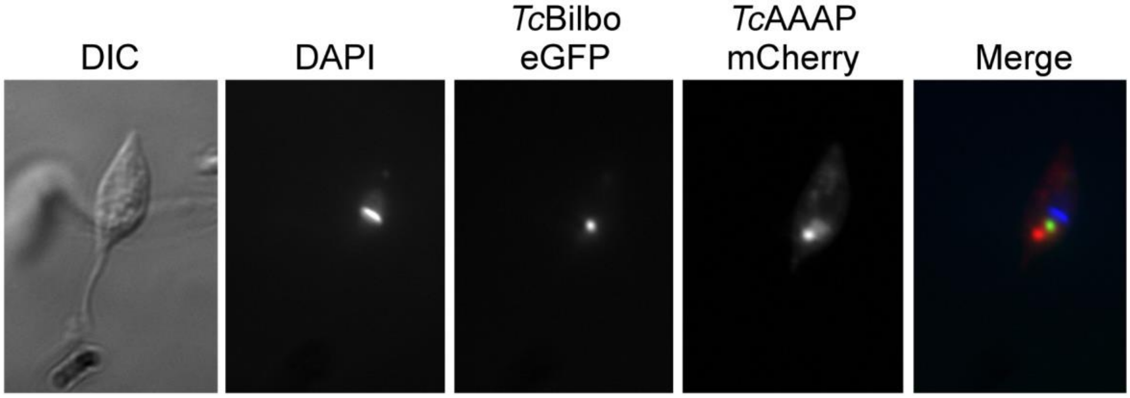

Supplementary Figure R4: Microphotography of MJ-Levin strain epimastigotes transfected with pTEXL-Neo harbouring a *Tc*Bilbo::eGFP fusion and pTEXL-Hyg containing an equivalent *Tc*AAAP::mCherry, after selection with 100 µg/mL of G418 and 500 µg/mL of hygromycin B.
