# Supplementary material for: Plasmid Vectors and Molecular Building Blocks for the Development of Genetic Manipulation Tools for Trypanosoma cruzi
Source: PLoS One. 2013 Oct 24;8(10):e80217. doi: 10.1371/journal.pone.0080217 (PMC3812015; doi:10.1371/journal.pone.0080217)
Supplement: Vector Information S1 — Extended restriction maps and general application guidelines. Detailed maps including restriction enzyme sites present in the MCSs of pTREXL, pTEXL, pTcR and pDIY are provided. For each vector, cloning strategies for the development of novel genetic manipulation tools or for the extension of already available genetic constructs are suggested. (PDF) [file pone.0080217.s003.pdf]

Additional vector information

1. pTREXL and pTEXL vectors

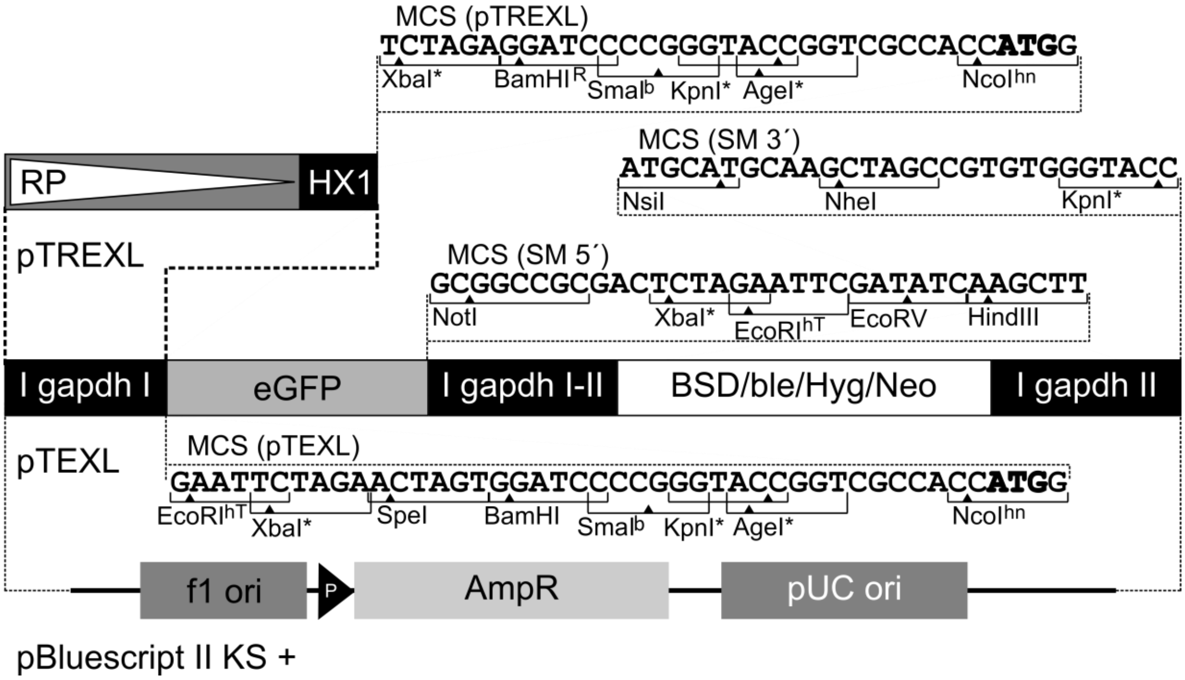

Figure S1. Expression vectors pTREXL and pTEXL. Extended restriction maps. The white box represents any of the four antibiotic resistance genes (BSD, blasticidin S deaminase; ble, bleomicin antibiotic family resistance gene; Hyg, hygromycin B phosphotransferase; Neo, aminoglycoside phosphotransferase). Black boxes are RNA processing sequences, the ones labelled I gapdh I, I gapdh I-II and I gapdh II correspond to ISs upstream, relative to and downstream from the g*GAPDH* tandem copy genes respectively. HX1 contains the ribosomal protein P2β splice acceptor site and the white triangle inside the grey box represents the ARN pol I ribosomal promoter (RP). Both vector series are identical except for the multiple cloning sites (MCS-pTREXL and MCS-pTEXL) and regulatory elements located upstream the enhanced green fluorescent protein coding sequence (grey box labelled eGFP). As indicated by the bold dotted lines the ribosomal promoter and HX1 splice acceptor sites present in the pTREXL vectors where replaced with the gapdh I IS in the derived pTEXL. The sequences of the respective MCSs as well as those on either side of the SM (MCS-SM 5´ and MCS-SM 3´) indicate available restriction sites. The NcoI sites contain the translation initiation codon for the eGFP gene (highlighted in bold). Restriction enzymes with superscripts have multiple recognition sites in: *, both series of vectors; R, pTREXL vectors; T, pTEXL vectors; and respectively b, h, n: BSD, Hyg or Neo gene harbouring vectors. Below the pBluescript II KS + phagemid used as backbone is schematized with the ampicillin resistance gene (AmpR) and beta lactamase promoter (black triangle labelled P), as well as the bacteriophage f1 (f1 ori) and pUC (pUC ori) bacterial replication origins.

2. p*Tc*R vectors

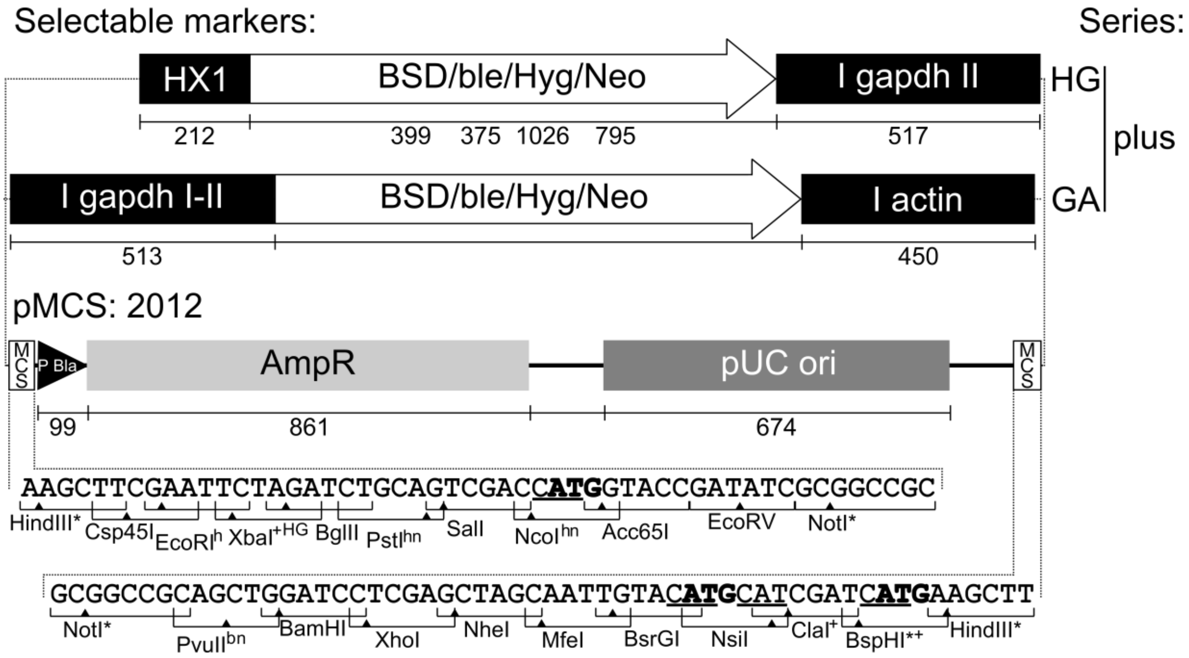

Figure S2. Schematic maps of p*Tc*R vector series. Extended restriction maps. For each of the four antibiotic resistance genes (BSD,ble,Hyg and Neo) two series of SMs were constructed. For the HG series, the ribosomal protein P2β splice acceptor site (HX1) with the IS downstream of the *GAPDH II* gene (I gapdh II) were placed at the 5´ and 3´ ends respectively. Analogously in the GA series, the coding sequences were inserted between the IS of the *GAPDH I* and *GAPDH II* genes (I gapdh I-II) and the IS relative to the *actin 1* and *actin 2* tandem copy genes (I actin). The p*Tc*R vector series were obtained cloning each SM at the unique HindIII restriction site of the minimal plasmid pMCS which is schematized with the multiple cloning sites (MCS), ampicillin resistance gene (AmpR), beta lactamase promoter (P Bla) and bacterial replication origin (pUC ori), below. Vectors with inserts in either orientation could be recovered. For clarity only those corresponding to the "plus" series are shown. Those in the equivalent "minus" series differ in that the SMs run in the opposite direction relative to the pMCS backbone. Each p*Tc*R vector was named according to the SM series (HG|GA), the harboured resistance gene, and insert orientation (+|-) (e.g. p*Tc*R-HG Neo +, p*Tc*R-GA ble - ,etc). The sequences of the MCSs, below the maps, show the available restriction enzyme sites. Potential translation initiation codons are indicated in bold (sense) or underlined (antisense). Enzymes with superscripts have multiple recognition sites in: *, any p*Tc*R vector; HG, the p*Tc*R-HG SM series; and respectively b, h, n: BSD, Hyg or Neo gene containing vectors. Additionally + indicates enzyme sites blocked by overlapping dam DNA methylation. The numbers correspond to the length in base pairs of each respective element above.

2.1. Restriction enzymes sites suitable for ligation of a p*Tc*R derived selectable marker

MCS	Compatible ends		MCS
NotI (*)<br>GC^GGCCGC	PspOMI<br>G^GGCCC		NotI (*)<br>GC^GGCCGC
EagI<br>C^GGCCG			EagI<br>C^GGCCG
EcoRV<br>GAT^ATC	Blunt		PvuII<br>CAG^CTG
Acc65I<br>G^GTACC	BsiWI<br>C^GTACG		BsrGI<br>T^GTACA
KpnI<br>GGTAC^C	---		---
NcoI (hn)<br>C^CATGG	PciI<br>A^CATGT		BspHI+ (*)<br>T^CATGA
SalI<br>G^TCGAC	AbsI<br>CC^TCGAGG		XhoI<br>C^TCGAG
PstI (hn)<br>CTGCA^G	SbfI<br>CCTGCA^GG		NsiI<br>ATGCA^T
BglII<br>A^GATCT	BclI+<br>T^GATCA		BamHI<br>G^GATCC
XbaI+ (HG)<br>T^CTAGA	AvrII<br>C^CTAGG	SpeI<br>A^CTAGT	NheI<br>G^CTAGC
---	---		BmtI<br>GCTAG^C
EcoRI (h)<br>G^AATTC	---		MfeI<br>C^AATTG
Csp45I<br>TT^CGAA	AclI<br>AA^CGTT	NarI<br>GG^CGCC	ClaI+<br>AT^CGAT
HindIII (*)<br>A^AGCTT	---		HindIII (*)<br>A^AGCTT

Table S1: The restriction enzyme sites in the MCSs of the p*Tc*R vector series are shown sharing a row with all the sites that can produce compatible ends with commercially available restriction enzymes. The sites have been sorted according to the order of appearance in the MCS that extends from the beta lactamase promoter to the selectable marker (left column). Although only one isoschizomer is indicated for each site, available neoschizomers are included below. The site of cleavage is indicated by ^. Restriction enzymes highlighted with + have a site blocked by overlapping dam DNA methylation. Those with round brackets have one additional site in: *, any p*Tc*R vector; HG the p*Tc*R-

HG selectable marker series; and respectively b, h, n: BSD, Hyg or Neo gene containing vectors.

2.2. Lengths of p*Tc*R vectors and derived selectable markers

R gene	p*Tc*R		SM	
	HG (+/-)	GA (+/-)	HG	GA
**BSD**	3141	3373	1135	1367
**ble**	3116	3348	1110	1342
**Hyg**	3767	4000	1761	1994
**Neo**	3563	3770	1530	1764

Table S2: The lengths in bp of the different p*Tc*R vectors are indicated according to the selectable marker series (HG/GA), insert orientation (+/-) and resistance gene (R gene). Additionally the length of the respective selectable markers produced as HindIII fragments are shown on the right. Particularly for those containing the Hyg resistance gene, adequate electrophoretic separation from the 2006 bp long backbone fragment might not be possible. Although various restriction enzymes can be used to break up pMCS remnants, possibly the best size discrimination can be obtained with *AseI*, *AclI* or, in the case of p*Tc*R-HG vectors, in addition to these *DraI*.

2.3. Suggested selectable marker cloning strategies

To illustrate ways in which already available genetic manipulation tools for *Trypanosoma cruzi* can be extended with p*Tc*R derived selectable markes, different cloning possibilities are provided in Tables S3, S4 and S5. These have been theoretically deduced according to reported sequences. The actual use and modification of the vectors should be done with permission of their respective authors.

2.3.1. Insertion of the selectable markers into pTEX (1), pRIBOTEX (2), pTREX (3) and derived vectors

The suggested replacement of the selectable marker originally developed for pTEX is based (at the 5´ insertion site) on restriction enzymes sites present in the 3´ region of the MCS (SalI/XhoI), remnants of the *gGAPDH I* gene (PstI) or de original gapdh I-II intergenic region (MfeI) and at the 3´ insertion site Csp45I, NheI or KpnI. Since the expression of the gene of interest relies on the gapdh I-II region the transfer should preferentially be done from p*Tc*R-GA vectors. In some cases this methodology might be performed on vectors already harbouring genes of interest.

Recipient s1	p*Tc*R-GA	Recipient s2
SalI/XhoI	**+ (dam)**	Csp45I

	SalI	ClaI+	
	**-**		
	XhoI	Csp45I	
	**+**		NheI
	SalI	NheI	
	**- (dam)**		
	XhoI	XbaI+	
	**+**		Acc65I (RP)
	SalI	BsrGI	
	**-**		Acc65I/KpnI (RP)
	XhoI	Acc65I/KpnI	
PstI (pTEX, pRIBOTEX)	**+ (dam)**		Csp45I
	PstI (hn)	ClaI+	
	**-**		
	NsiI	Csp45I	
	**+**		NheI
	PstI (hn)	NheI	
	**- (dam)**		
	NsiI	XbaI+	
	**+**		Acc65I (RP)
	PstI (hn)	BsrGI	
	**-**		Acc65I/KpnI (RP)
	NsiI	Acc65I/KpnI	
MfeI (pTEX)	**+ (dam)**		Csp45I
	EcoRI (h)	ClaI+	
	**-**		
	MfeI	Csp45I	
	**+**		NheI
	EcoRI (h)	NheI	
	**- (dam)**		
	MfeI	XbaI+	
	**+**		Acc65I (RP)
	EcoRI (h)	BsrGI	
	**-**		Acc65I/KpnI (RP)
	MfeI	Acc65I/KpnI	

Table S3: Restriction enzymes required to digest the recipient vectors at either side of the selectable marker to be replaced are shown in the left and right columns. For any combination the respective restriction enzymes required to produce a fragment containing a p*Tc*R-GA selectable marker suitable for ligation are indicated in the middle columns. In each case the orientation series are shown (+/-) as well as the requirement to derive the

p*Tc*R vectors from DNA methylase deficient bacteria (dam). Vector names in round brackets have additional restriction enzymes in inadequate regions of the molecule and consequently the cloning step is likely to require partial digestion. The interfering PstI site in the MCS of pTEX and pRIBOTEX vectors can be removed by insertion of the gene of interest. RP represents ribosomal promoter containing vectors (pRIBOTEX and pTREX). Restriction enzymes highlighted with + have a site blocked by overlapping dam DNA methylation, or have additional sites in respectively Hyg (h) or Neo (n) gene containing vectors.

2.3.2. Insertion of the selectable markers into pTcINDEX (4)

The selectable marker replacement based on digestion of pTcINDEX with XbaI or PstI will preserve the original gapdh I intergenic sequence included in this vector. Any p*Tc*R derived selectable marker can be inserted downstream, however if a p*Tc*R-HG vector is used as donor and digested with XbaI, the HX1 region will be lost and consequently the resistance gene will rely on the gapdh I region for expression. On the other hand if MfeI is used to prepare pTcINDEX only a fragment of gapdh I will remain. Since p*Tc*R-GA ble and p*Tc*R-GA Hyg vectors contain a SpeI site between the resistance gene and the actin intergenic sequence, insertion of these selectable markers will interfere with adequate vector linearization prior to transfection.

<table>
<tr><th>pTcINDEX s1</th><th colspan="2">pTcR</th><th>pTcINDEX s2</th></tr>
<tr><td rowspan="9">XbaI</td><td colspan="2">HG+ (dam)</td><td rowspan="13">Csp45I</td></tr>
<tr><td>XbaI (HX1-)</td><td>ClaI+</td></tr>
<tr><td colspan="2">HG-</td></tr>
<tr><td>XbaI (HX1-)</td><td>Csp45I</td></tr>
<tr><td>NheI</td><td>Csp45I</td></tr>
<tr><td colspan="2">GA+ (dam)</td></tr>
<tr><td>XbaI+</td><td>ClaI+</td></tr>
<tr><td colspan="2">GA-</td></tr>
<tr><td>NheI</td><td>Csp45I</td></tr>
<tr><td rowspan="8">MfeI</td><td colspan="2">HG+/GA+ (dam)</td></tr>
<tr><td>EcoRI (h)</td><td>ClaI+</td></tr>
<tr><td colspan="2">HG-/GA-</td></tr>
<tr><td>MfeI</td><td>Csp45I</td></tr>
<tr><td colspan="2">HG+/GA+</td><td rowspan="9">PstI</td></tr>
<tr><td>EcoRI (h)</td><td>NsiI</td></tr>
<tr><td colspan="2">HG-/GA-</td></tr>
<tr><td>MfeI</td><td>PstI (hn)</td></tr>
<tr><td rowspan="5">XbaI</td><td colspan="2">HG+</td></tr>
<tr><td>XbaI (HX1-)</td><td>NsiI</td></tr>
<tr><td colspan="2">HG-</td></tr>
<tr><td>XbaI (HX1-)</td><td>PstI (hn)</td></tr>
<tr><td>NheI</td><td>PstI (hn)</td></tr>
</table>

	**GA+ (dam)**		
	XbaI+	NsiI	
	**GA-**		
	NheI	PstI (hn)	
PstI	**HG+/GA+ (↔)**		
	PstI (hn)	NsiI	
	**HG-/GA- (↔)**		
	NsiI	PstI (hn)	

Table S4: Restriction enzymes required to digest pTcINDEX at either side of the selectable marker to be replaced are shown in the left and right columns. For any combination the respective restriction enzymes required to produce a fragment containing a p*Tc*R selectable marker suitable for ligation are indicated in the middle columns. In each case the vector series are shown (HG/GA and +/-) as well as the requirement to derive the p*Tc*R vectors from DNA methylase deficient bacteria (dam). A double arrow (↔) indicates that after digestion the recipient vectors requires treatment with phosphatases and that the ligation will produce vectors with inserts in both orientations. Restriction enzymes highlighted with + have a site blocked by overlapping dam DNA methylation, or have additional sites in respectively Hyg (h) or Neo (n) gene containing vectors. Those indicated with HX1- will generate a HG selectable marker missing the HX1 fragment.

2.3.3. Insertion of the selectable markers into p*Tc*GW vectors (5)

Since the digestion of p*Tc*GW with SpeI implies the loss of a TcUI intergenic sequence, in this case the p*Tc*R-GA should be employed to provide a selectable marker with a suitable 3´ UTR for expression of the gene of interest.

**p*Tc*GW s1**	**p*Tc*R**		**p*Tc*GW s2**
SpeI	**GA+ (dam)**		Acc65I (RP)
	XbaI+	BsrGI	
	**GA-**		Acc65I/KpnI (RP)
	NheI	Acc65I/KpnI	
	**GA+ (dam)**		XhoI (T)
	XbaI+	XhoI	
	**GA-**		
	NheI	SalI	
XhoI (T)	**HG+/GA+ (↔)**		
	SalI	XhoI	
	**HG-/GA- (↔)**		
	XhoI	SalI	
	**HG+/GA+**		Acc65I (RP)
	SalI	BsrGI	
	**HG-/GA-**		Acc65I/KpnI (RP)

	XhoI	Acc65I/KpnI	

Table S5: Restriction enzymes required to digest p*Tc*GW vectors at either side of the selectable marker to be replaced are shown in the left and right columns. For any combination the respective restriction enzymes required to produce a fragment containing a p*Tc*R selectable marker suitable for ligation are indicated in the middle columns. In each case the vector series are shown (HG/GA and +/-) as well as the requirement to derive the p*Tc*R vectors from DNA methylase deficient bacteria (dam). A double arrow (↔) indicates that after digestion the recipient vectors requires treatment with phosphatases and that the ligation will produce vectors with inserts in both orientations. Restriction enzymes highlighted with + have a site blocked by overlapping dam DNA methylation, or have additional sites in respectively Hyg (h) or Neo (n) gene containing vectors, p*Tc*TAPN (T) or in the ribosomal promoter (RP) of any p*Tc*GW.

2.3.4. Conversion of p*Tc*R vectors into Gateway entry plasmids

The easiest way to produce suitable entry plasmids for Gateway based transfer of HG and GA selectable markers involves pCR8/GW/TOPO TA Cloning Kit (Invitrogen). Although each selectable marker can be PCR amplified with primer pairs similar to HX1HF-G2HR for the HG series or GHF-AHR for GA (see supplementary material and methods) and cloned into pCR8/GW/TOPO, an alternative that avoids the requirement of primers is available. Fragments containing any p*Tc*R harboured selectable markers can be produced by digestion of both MCS on either side with restriction enzymes that generate 5´ protruding or blunt ends (e.g. HindIII). These segments can then be treated with Taq DNA polymerase filling in the sites and transferring the required 3´ adenosine residue. With both alternatives, after insertion into pCR8/GW/TOPO the clones have to be screened for appropriate insert orientation. The resulting entry plasmids can be employed for the generation of TGRV in a methodology similar to the one previously reported (6) except for the fact that in this case the resistance genes are regulated by constitutive intergenic regions.

3. pDIY vectors

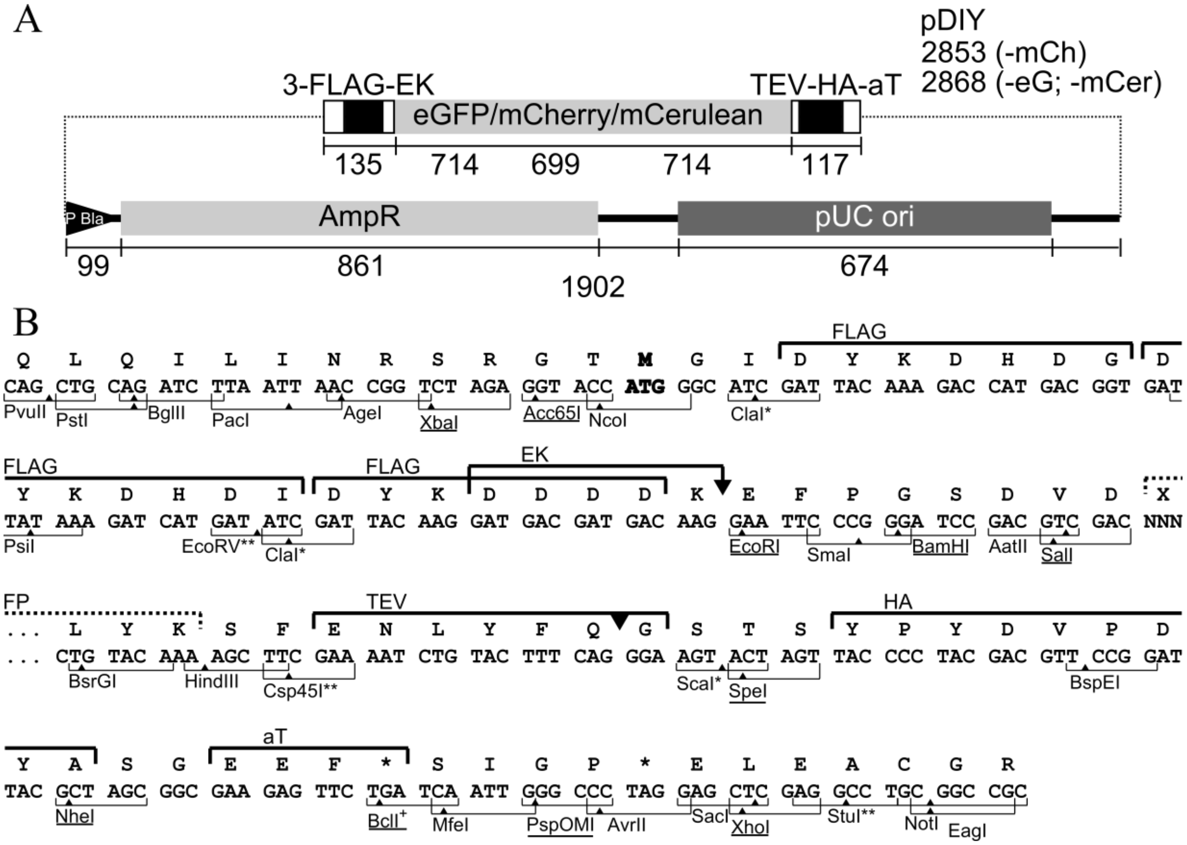

Figure S3. pDIY vectors. (A) Schematic map: The main feature of pDIY vectors is the coding sequence represented above. The white segments indicate at least three consecutive restriction enzyme sites while the black rectangles correspond to coding sequences for, at the 5´ end, three FLAG epitopes (3-FLAG) with an overlapping enterokinase cleavage site (EK) and at the 3´ end, the tobacco etch virus protease cleavage site (TEV), the human influenza hemagglutinin (HA) and alpha tubulin (aT) epitopes. All pDIY vectors are identical except for the fluorescent protein coded in each case (grey rectangle). The currently three available variants, with enhanced green (eGFP), monomeric red (mCherry) and monomeric cyan (mCerulean) fluorescent protein coding sequences were named pDIY-eG, pDIY-mCh and pDIY-mCer respectively. The scheme below depicts the beta lactamase promoter (P Bla) the ampicillin resistance gene (AmpR) and the bacterial replication origin (pUC ori) of the minimal plasmid in which each coding sequence was inserted. The numbers are the lengths, in base pairs, of the respective element above, the minimal plasmid or each pDIY vector (top right). (B) The complete coding sequence of pDIY vectors shows the reading frame, predicted translation product and stop codons (*). For clarity most of the fluorescent protein coding sequences (FP) were omitted (dotted line). The solid lines indicate the extension of the three individual

FLAG and the single HA and aT epitopes as well as the EK and TEV protease recognition sequences. The black triangles, in the latter two, point to the cleavage sites. Restriction enzymes for the available restriction sites are shown below the nucleotide sequence. The *NcoI* site contains the translation initiation codon (highlighted in bold). Enzymes with superscripts have multiple sites in: *, any pDIY vector; **, only in pDIY-mCh. Due to DNA methylation the *BclI* site is unavailable unless the plasmid is derived from dam- bacteria strains, as indicated by +. Underlined names highlight in frame 4-base 5´ protruding end restriction enzyme sites.

3.1. Restriction enzymes sites suitable for ligation of pDIY vectors

pDIY	Frame	Compatible ends		
PvuII<br>`CAG^CTG`	1	Blunt		
PstI<br>`CTGCA^G`	1	NsiI<br>`ATGCA^T`	SbfI<br>`CCTGCA^GG`	
BglII<br>`A^GATCT`	2	BamHI<br>`G^GATCC`	BclI+<br>`T^GATCA`	
PacI<br>`TTAAT^TAA`	1	PvuI<br>`CGAT^CG`	AsiSI<br>`GCGAT^CGC`	
AgeI<br>`A^CCGGT`	2	BspEI<br>`T^CCGGA`	NgoMIV<br>`G^CCGGC`	XmaI<br>`C^CCGGG`
XbaI<br>`T^CTAGA`	1	AvrII<br>`C^CTAGG`	NheI<br>`G^CTAGC`	SpeI<br>`A^CTAGT`
Acc65I<br>`G^GTACC`	1	BsiWI<br>`C^GTACG`	BsrGI<br>`T^GTACA`	
KpnI<br>`GGTAC^C`	1	---		
NcoI<br>`C^CATGG`	2	PciI<br>`A^CATGT`	BspHI<br>`T^CATGA`	
ClaI (*)<br>`AT^CGAT`	1	Csp45I<br>`TT^CGAA`	AclI<br>`AA^CGTT`	NarI<br>`GG^CGCC`
PsiI<br>`TTA^TAA`	3	Blunt		
EcoRV (**)<br>`GAT^ATC`	1	Blunt		
EcoRI<br>`G^AATTC`	1	MfeI<br>`C^AATTG`		
XmaI<br>`C^CCGGG`	3	AgeI<br>`A^CCGGT`	BspEI<br>`T^CCGGA`	NgoMIV<br>`G^CCGGC`
SmaI<br>`CCC^GGG`	3	Blunt		
BamHI	1	BglII	BclI+	

G^GATCC		A^GATCT	T^GATCA	
AatII<br>GACGT^C	1	---		
ZraI<br>GAC^GTC	1	Blunt		
SalI<br>G^TCGAC	1	XhoI<br>C^TCGAG	AbsI<br>CC^TCGAGG	
BsrGI<br>T^GTACA	2	Acc65I<br>G^GTACC	BsiWI<br>C^GTACG	
HindIII<br>A^AGCTT	3	---		
Csp45I (**)<br>TT^CGAA	1	ClaI<br>AT^CGAT	AclI<br>AA^CGTT	NarI<br>GG^CGCC
ScaI (*)<br>AGT^ACT	1	Blunt		
SpeI<br>A^CTAGT	1	XbaI<br>T^CTAGA	AvrII<br>C^CTAGG	NheI<br>G^CTAGC
BspEI<br>T^CCGGA	3	AgeI<br>A^CCGGT	NgoMIV<br>G^CCGGC	XmaI<br>C^CCGGG
NheI<br>G^CTAGC	1	SpeI<br>A^CTAGT	XbaI<br>T^CTAGA	AvrII<br>C^CTAGG
BmtI<br>GCTAG^C	1	---		
BclI+<br>T^GATCA	1	BamHI<br>G^GATCC	BglII<br>A^GATCT	
MfeI<br>C^AATTG	2	EcoRI<br>G^AATTC		
ApaI<br>GGGCC^C	1	---		
PspOMI<br>G^GGCCC	1	EagI<br>C^GGCCG	NotI<br>GC^GGCCGC	
AvrII<br>C^CTAGG	2	NheI<br>G^CTAGC	SpeI<br>A^CTAGT	XbaI<br>T^CTAGA
SacI<br>GAGCT^C	1	---		
Eco53kI<br>GAG^CTC	1	Blunt		
XhoI<br>C^TCGAG	1	SalI<br>G^TCGAC	AbsI<br>CC^TCGAGG	
StuI (**)<br>AGG^CCT	2	Blunt		

NotI<br>GC^GGCCGC	2	EagI<br>C^GGCCG	PspOMI<br>G^GGCCC
EagI<br>C^GGCCG	3	NotI<br>GC^GGCCGC	PspOMI<br>G^GGCCC

Table S6: The restriction enzyme sites in the pDIY vector series are shown sharing a row with all the sites that can produce compatible ends with commercially available restriction enzymes. The sites have been sorted according to the order of appearance from the beta lactamase promoter, through the coding sequence to the replication origin (left column). Although only one isoschizomer is indicated for each site, available neoschizomers are included below. The site of cleavage is indicated by ^. Restriction enzymes highlighted with + have a site blocked by overlapping dam DNA methylation. Those with round brackets have one additional site in: *, any pDIY vector; **, the pDIY-mCh. The middle column indicates the translation frame at the first residue of the site.

3.2. pDIY coding sequence transformations

#		Coding sequence	Restriction enzyme<br>5´	 3´	DNA pol.	Site reg.
0		M-2F-FEK-FP-TEV-HA-αT*	---	---	---	---
1	a	M-FEK-FP-TEV-HA-αT*	ClaI		---	ClaI
	b		NcoI	EcoRV	+	---
2	a	M-FP-TEV-HA-αT*	ClaI*	EcoRI	+	EcoRI
	b		ClaI*	BamHI	+	BamHI
	c		ClaI*	SalI	+	SalI
	d		NcoI	XmaI	+	---
	e		NcoI	ZraI	+	NcoI
3#	a	M-2F-FP-TEV-HA-αT*	EcoRV	XmaI	+	---
	b		EcoRV	ZraI	---	---
4		M-2F-FEK	SalI	XhoI	---	---
5		M-2F-FEK-FP*-TEV-HA-αT*	HindIII		+	NheI
6	a	M-TEV-HA-αT*	ClaI*	Csp45I	---	---
	b		NcoI	HindIII	+	---
7	a	M-2F-FEK-TEV-HA-αT*	EcoRI	Csp45I	+	EcoRI
	b		BamHI	Csp45I	+	BamHI
	c		SalI	Csp45I	+	SalI
8#		M-2F-FEK-FP-HA-αT*	Csp45I	SpeI	+	---
9#		M-2F-FEK-FP-αT*	Csp45I	NheI	+	NheI
10		M-2F-FEK-FP-TEV-αT*	SpeI	NheI	---	---
11		M-2F-FEK-FP-TEV-(HA)$_n$-αT*	I:X	I:NheI	---	---
		↓				
		M-2F-FEK-FP-TEV-(HA)$_{2n}$-αT*	V:X	V:SpeI		
12		M-2F-FEK-FP-TEV-(HA)$_n$-αT*	BspEI		---	BspEI

		↓<br>M-2F-FEK-FP-TEV-$(HA)_1$-αT*				
13		HA-aT*	XbaI	SpeI	---	---
14		M-HA-aT*	NcoI	ScaI*	+	---
15		aT*	XbaI	NheI	---	---
16		M-aT*	NcoI	BspEI	+	---
17		M-2F-FEK-FP	SpeI	AvrII	---	---
18		M-2F-FEK-FP-TEV	NheI	AvrII	---	---
19	a	M-2F-FEK-FP*	SpeI	AvrII	+	---
	b		Csp45I	PspOMI	+	PspOMI
20		M-2F-FEK-FP-TEV*	NheI	AvrII	+	NheI
21		FP-TEV-HA-αT*	BglII	BamHI	---	---
22	a	M-2F-FEK*	BamHI	BclI+	---	---
	b		EcoRI	AvrII	+	EcoRI
	c		BamHI	AvrII	+	BamHI
	d		SalI	AvrII	+	SalI
23		M-2F-TEV-HA-αT*	EcoRV	HindIII	+	---

Table S7: With the original coding sequence (0) as a starting point, the DNA manipulation steps required to obtain the different derivatives enumerated on the left are indicated. The last step in each case involves fragment ligations or plasmid recircularization. Needed restriction enzymes as well as treatment with DNA polymerases (DNA pol.; + or ---) are shown. In some cases restriction sites might be regenerated (Site reg.), and thus can be used to evaluate the success of the step. Cases highlighted with # imply partial *EcoRV* or *Csp45I* digestion when performed on pDIY-mCh. Transformation 11 involves an insert (I) and vector (V) fragment which can be derived from any pDIY vector. X represents any restriction enzyme with a site present preferentially upstream the fluorescent protein gene. For clarity the coding sequence for each element is represented by an abbreviation: M, translation initiation codon inside the *NcoI* site; 2F, the two initial FLAG copies of the 3-FLAG epitope; FEK, third FLAG copy that partially overlaps the EK recognition site; FP, any of the three available fluorescent protein genes (eGFP, mCherry and mCerulean); TEV, tobacco etch virus protease recognition site; HA, $(HA)_n$, $(HA)_{2n}$ and $(HA)_1$, are different numbers of tandem copies coding the HA epitope; αT, alpha tubulin epitope; *, translation stop codon. Restriction enzymes highlighted with + have a site blocked by overlapping dam DNA methylation and those indicated with * have one additional recognition sites in any pDIY vector.

3.3. Suggested strategies for the transference of pDIY-harboured coding sequences into already available *Trypanosoma cruzi* genetic manipulation tools

Considering ways to produce flexible plasmids, the examples outlined below focus on the insertion, into the different target vectors, of unmodified, full length coding sequences. The resulting molecules can later be modified according to the steps indicated in Table S8. Alternatively fragments of the coding sequences can be transferred instead or modified in advance.

3.3.1. Transfer into pTEX (1), pRIBOTEX (2), pTREX (3) and derived vectors

In order to preserve available coding sequence transformation possibilities, a good cloning strategy consists in removing as many restriction enzyme sites as possible, from the target vector, during the cloning step. For pTEX and pTREX this can be done cloning the pDIY derived insert as an XbaI-XhoI fragment into the target vectors digested with the same enzymes. For pRIBOTEX, a similar methodology might be employed except that the recipient should be digested with BamHI and XhoI and the insert from pDIY should be obtained as a BglII-XhoI fragment.

3.3.2. Transfer into pTcINDEX (4)

To remove most of the MCS, pTcINDEX can be digested with NgoMIV (a NaeI neoschizomer) and the blunt end restriction enzyme NruI. The obtained linear molecule can then be directionally ligated to the pDIY derived coding sequence produced as a compatible cohesive and blunt ended fragment with AgeI-StuI. An important aspect to consider is the fact that the resulting molecule will contain an additional SpeI site that might interfere with vector linearization prior to transfection. To overcome this limitation the chosen pDIY can be subjected to a modification in advance to remove the contained SpeI site (transformations 8, 9 and 10 of Table S7 for example). Alternatively the SpeI site of pTcINDEX can be replaced with an equivalent for a rare cutting restriction enzyme (other than NotI) by ligation of an oligonucleotide linker (in the case of SwaI: `CTAGATTTAAAT`).

3.3.3. Transfer into p*Tc*GW vectors (5) for N-terminal fusions

As in the pDIY vector series the translation initiation codon of p*Tc*GW vectors resides inside a NcoI site, however, since the latter contain additional recognition sites, direct insertion of the coding sequence might require difficult partial digestions. Nevertheless, these can be avoided employing a two-step cloning strategy. For the first step the coding sequence insert is produced from pDIY as an NcoI-XhoI fragment. Insertion into any pTcGW digested with the same enzymes will remove the original coding sequence, Gateway cassette and selectable marker producing the intermediate vector pTc-I. The second step involves an XbaI-ApaI segment containing the Gateway cassette and selectable marker derived from a p*Tc*GW. There are two ways in which to ligate this fragment into pTc-I. If the recipient is digested with SpeI-ApaI the resulting vector (p*Tc*GW-Nt1) will be missing the coding sequence for the HA and aT epitopes as well as the stop codon. Instead, if the digestion on p*Tc*-I is performed with NheI and ApaI, after ligation the resulting p*Tc*GW-Nt2 vector will be only lacking the sequences relative to the aT epitope and translation stop codon.

3.3.4. Transfer into p*Tc*GW vectors (5) for C-terminal fusions

The conversion of any p*Tc*GW into a Gateway based expression vector for C-terminal fusions implies the elimination of the original traceable element coding sequences as well as translation initiation codon. This can be performed by PCR amplification of the ribosomal promoter and first TcUI intergenic sequence on any p*Tc*GW as template with

the universal M13F primer (GTTTTCCCAGTCACGACGTTGTA) and a reverse primer specific for TcUI which incorporates an XbaI site (e.g.: TTTTCTAGAGGTTTCACGTTCCTAACAGTGTGT). The amplification product can be digested with SacI and XbaI and inserted into any p*Tc*GW prepared with the same enzymes generating the intermediate vector p*Tc*GW-I. On the other hand the translation frame of the coding sequence harboured in any pDIY vector requires adjustment. Such a modified plasmid (pDIY-A) can be obtained by digestion with Acc65I, treatment with DNA polymerases and religation. The success of the step can be evaluated by loss of the KpnI/Acc65I site or SnaBI generation and should be confirmed by sequencing. The final step implies the transfer of the adjusted coding sequence of pDIY-A, produced as a XbaI-AvrII segment, to p*Tc*GW-I digested with SpeI and treated with phosphatases. The obtained clones should be screened for proper insert orientation.

3.4. pDIY derived coding sequence transformations after incorporation into already available *Trypanosoma cruzi* genetic manipulation tools

#		Restriction enzyme 5´	Restriction enzyme 3´	pTEX	pRIBOTEX	pTREX	pTcINDEX	p*Tc*GW Nt1	p*Tc*GW Nt2	p*Tc*GW Ct
1	a	ClaI		+	+	+	+	+	+	+
	b	NcoI	EcoRV	PD(2;1)	PD(2;1)	PD(2;1)	PD(2;1)	PD(3;2)	PD(3;2)	PD(3;2)
2	a	ClaI	EcoRI	PD(2;2)	+	+	PD(2;5)	PD(2;2) GW	PD(2;2) GW	PD(2;2) GW
	b	ClaI	BamHI	+	+	PD(2;2)	PD(2;1)	PD(2;3) GW	PD(2;3) GW	PD(2;3) GW
	c	ClaI	SalI	+	+	+	+	PD(2;2) GW	PD(2;2) GW	PD(2;2) GW
	d	NcoI	XmaI	PD(2;1)	PD(2;1)	PD(2;1)	PD(2;3)	PD(3;2)	PD(3;2)	PD(3;2)
	e	NcoI	ZraI	PD(2;1)	PD(2;1)	PD(2;1)	PD(2;3)	PD(3;1)	PD(3;1)	PD(3;1)
3#	a	EcoRV	XmaI	+	+	+	PD(1;3)	PD(2;2)	PD(2;2)	PD(2;2)
	b	EcoRV	ZraI	+	+	+	PD(1;3)	PD(2;1)	PD(2;1)	PD(2;1)
4		SalI	XhoI	+	+	+	PD(1;2)	NA	NA	NA
5		HindIII		+	+	+	PD(2)	+	+	+
6	a	ClaI	Csp45I	PD(2;2)	PD(2;2)	PD(2;2)	PD(2;2)	+	+	+
	b	NcoI	HindIII	PD(2;1)	PD(2;1)	PD(2;1)	PD(2;2)	PD(3;1)	PD(3;1)	PD(3;1)
7	a	EcoRI	Csp45I	PD(2;2)	PD(1;2)	PD(1;2)	PD(5;2)	PD(2;1) GW	PD(2;1) GW	PD(2;1) GW
	b	BamHI	Csp45I	PD(1;2)	PD(1;2)	PD(2;2)	PD(1;2)	PD(3;1) GW	PD(3;1) GW	PD(3;1) GW
	c	SalI	Csp45I	PD(1;2)	PD(1;2)	PD(1;2)	PD(1;2)	PD(2;1) GW	PD(2;1) GW	PD(2;1) GW
8#		Csp45I	SpeI	PD(2;1)	PD(2;1)	PD(2;1)	PD(2;2)	NA	PD(2;2)	+
9#		Csp45I	NheI	PD(2;2)	PD(2;2)	PD(2;2)	PD(2;4)	NA	NA	+
10		SpeI	NheI	PD(1;2)	PD(1;2)	PD(1;2)	PD(2;4)	NA	NA	+
11		I:X	I:NheI	+	+	+	+	NA	+	+
		V:X	V:SpeI				PD(2)		PD(2)	
12		BspEI		+	+	+	PD(5)	NA	PD(2) GW	PD(2) GW

13		XbaI	SpeI	+	+	+	PD(2;2)	NA	NA	NA
14		NcoI	ScaI*	PD(2;2)	PD(2;2)	PD(2;2)	PD(2;3)	NA	PD(3;3)	PD(3;3)
15		XbaI	NheI	PD(1;2)	PD(1;2)	PD(1;2)	PD(2;4)	NA	NA	NA
16		NcoI	BspEI	PD(2;1)	PD(2;1)	PD(2;1)	PD(2;5)	NA	PD(3;2)	PD(3;2)
17		SpeI	AvrII	+	+	+	PD(2;1)	NA	NA	NA
18		NheI	AvrII	PD(2;1)	PD(2;1)	PD(2;1)	PD(4;1)	NA	NA	NA
19	a	SpeI	AvrII	+	+	+	PD(2;1)	NA	NA	NA
	b	Csp45I	PspOMI	PD(2;1)	PD(2;1)	PD(2;1)	PD(2;1)	NA	NA	PD(1;2)
20		NheI	AvrII	PD(2;1)	PD(2;1)	PD(2;1)	PD(4;1)	NA	NA	NA
21		BglII	BamHI	NA	NA	NA	NA	NA	NA	NA
22	a	BamHI	BclI	+	+	PD(2;1)	+	NA	NA	+
	b	EcoRI	AvrII	PD(2;1)	+	+	PD(5;1)	NA	NA	NA
	c	BamHI	AvrII	+	+	PD(2;1)	+	NA	NA	NA
	d	SalI	AvrII	+	+	+	+	NA	NA	NA
23		EcoRV	HindIII	+	+	+	PD(1;2)	PD(2;1)	PD(2;1)	PD(2;1)

Table S8: The feasibility of obtaining the derived molecules of Table S7 after incorporation of the pDIY harboured coding sequences into already available *Trypanosoma cruzi* genetic manipulation tools is shown. As a reference the required restriction enzymes are included on the left. Easily performed steps involving unique restriction sites are indicated with +. PD represents cases where additional sites imply partial digestions. To provide a means to estimate the difficulty of such a methodology the number of the involved single or respective 5´ and 3´ restriction enzyme sites are indicated inside the round brackets. Methylation blocked sites have been omitted. In some cases partial digestion steps can be avoided for the modified p*Tc*GW if performed after Gateway recombination (indicated with GW) unless the cloned sequence contains additional sites. Some transformations are unavailable (NA).

4. General guidelines for the construction of p*Tc*R and pDIY based targeted gene replacement vectors (TGRVs) and episomal expression vectors (EEVs)

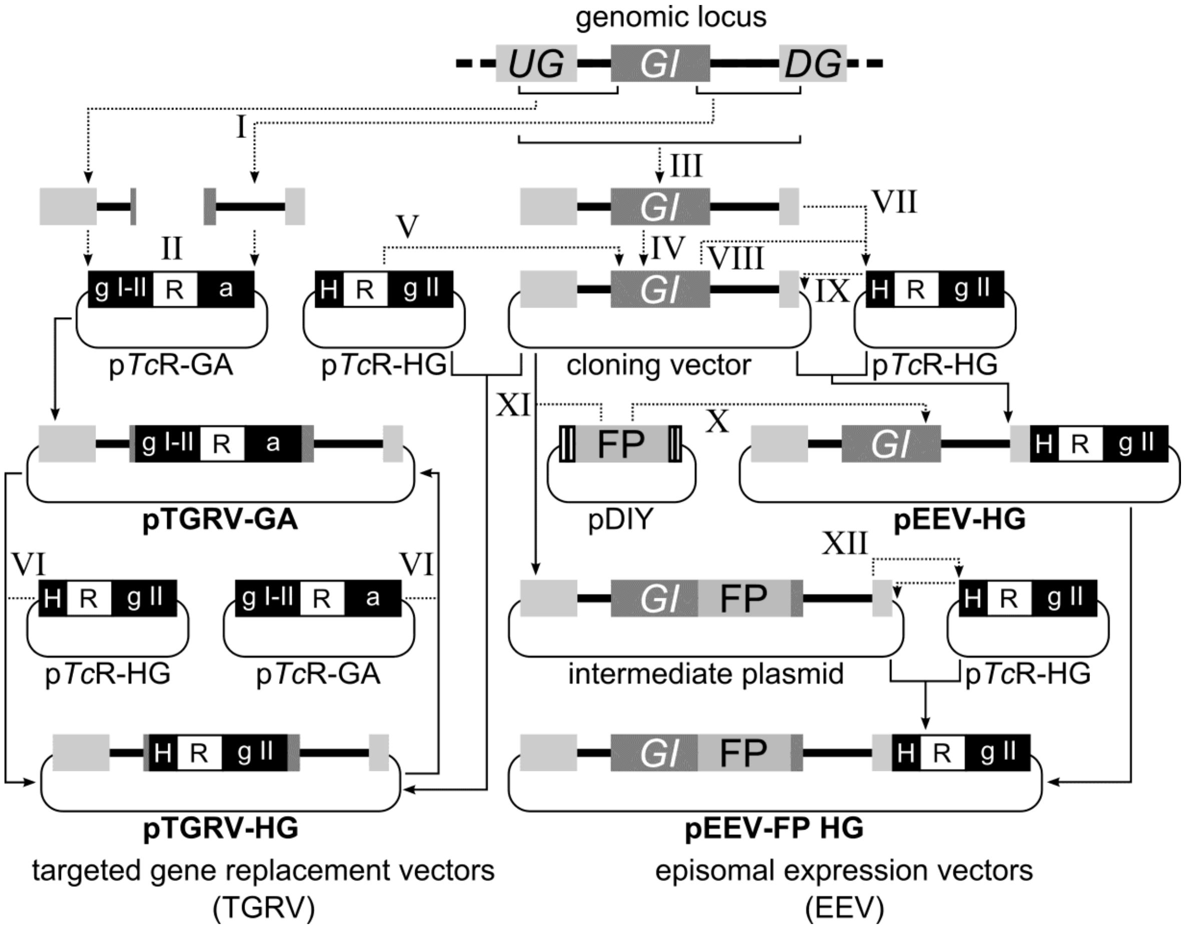

Figure S4. Diagram showing potential strategies for the construction of specific genetic manipulation tools with the building blocks developed in this work. The left and right sides respectively focus on the generation of TGRV or EEV for a hypothetical gene of interest (*GI*). The genomic locus of this gene is schematized above with flanking genes upstream (*UG*) and downstream (*DG*) as well as ISs (solid thick lines). Plasmids are represented as ovals and for clarity all the elements required for propagation in bacteria have been omitted. The SMs of p*Tc*R vectors are shown simplified with black sections labelled H, g I-II, g II and a corresponding, respectively, to the ISs HX1, I gapdh I-II, I gapdh II and I actin in Figure S2. The white box R represents any of the five antibiotic resistance genes employed in this work. Similarly for the pDIY vector only the presence of the fluorescent protein (FP) coding sequence is indicated. Apart from the developed plasmids and fragments derived from *T. cruzi* genome, the only optional element involved in the strategies outlined is a standard cloning vector. Solid arrows point to resulting constructs and those with names highlighted in bold can be directly used in the generation of recombinant parasite strains. Dotted arrows with roman numerals signal specific construction steps.

4.1 Construction of targeted gene replacement vectors.

The p*Tc*R series are particularly well suited for the development of TGRVs. These can be obtained in different ways (Figure S4). The simplest alternative consists in isolating regions flanking the target gene by PCR with primers that incorporate adequate restriction enzyme sites into the amplification products (I). Afterwards, these fragments can be sequentially cloned into any p*Tc*R vector on either side of the SM with restriction enzyme based techniques (II). Even though the multiple steps might be time consuming this strategy only requires universally available DNA manipulation reagents and equipment, it therefore constitutes the best and cost efficient option when few specific TGRVs are needed. On the other hand, for large scale projects the p*Tc*R vectors can be used according to a high-throughput scheme. Using the In-Fusion (7) (Clontech) vaccinia virus DNA polymerase based seamless cloning system (8), both targeting sequences can simultaneously be inserted into double digested p*Tc*R vectors in a single step. For this strategy the amplification products require on either end ≈15 bp extensions homologous to regions of the p*Tc*R MCSs and consequently are likely to contain restriction enzyme sites. This implies that upon unsuccessful In-Fusion cloning, standard methods are still available options. Another way to adapt the p*Tc*R vectors for high-throughput methodologies, consists in transferring the SMs to the pCR8/GW/TOPO (Invitrogen) PCR fragment cloning plasmid (see 2.3.4). The resulting Gateway entry vectors can extend the already available Multisite-Gateway (Invitrogen) (9) based high-throughput system for gene knockout in *Trypanosoma cruzi* (6). In addition, if the gene to be targeted contains restriction enzyme sites compatible with those in the MCSs of the p*Tc*R vectors an alternative strategy might be employed. A section of the genomic locus including the subject gene and flanking regions can be PCR-amplified as a single fragment (III) and inserted in an appropriate cloning vector (IV). Later on, a TGRV can be produced interrupting or replacing a portion of the coding sequence by insertion of any p*Tc*R harboured SM (V). In general, the additional restriction enzymes sites acquired by the construct, facilitate subsequent exchanges of these elements (VI).

To estimate the number of genes susceptible to this approach all the predicted genomic coding sequences of the reference CL-Brener strain (10) were scanned for the presence of suitable cohesive end restriction enzyme sites. Even though 97.2 % of the sequences contain at least one, in some cases these reside at inadequate locations, overlap blocking methylation motifs or coexist with multiple identical sites in other regions of the gene. Thus, the possibility of exploiting the presence of different restriction enzyme sites in subject sequences requires evaluation on a case-by-case basis. For assistance in this task the results of the analysis can be obtained upon request.

4.2. Construction of episomal expression vectors.

Regardless of the presence of appropriate restriction enzyme sites, genomic loci segments can be subject of another interesting methodology. Inserting the amplification product directly into a p*Tc*R vector (VII), or if already available in a cloning plasmid, by adequate subcloning of fragments to the respective MCSs (VIII or IX) an EEV can be obtained. Although after transfection the expression levels of the transgene are likely to differ from those of the endogenous counterparts, much of the stage specific regulation might be preserved. During the study or generation of null mutant strains, this strategy constitutes an attractive alternative for the construction of phenotype rescuing vectors for functional complementation (11). Furthermore, in some cases TGRVs might share the same construction intermediates needed to generate the associated EEVs.

4.3. Construction of traceable element expressing episomal expression vectors.

Given the simple EEV construction approach an ever increasing number of novel expression vectors could easily be produced. Depending on the particular features detected in these, some are likely to become starting points for the development of additional improved genetic manipulation tools. To broaden the range of applications the first step towards construct refinement might consist in replacing the *GI* with MCSs or traceable elements. The pDIY vectors developed in this work are particular good sources for the latter. In some cases where the coding sequences contain adequately placed compatible end restriction enzyme sites, it could be possible to transfer the given elements from the pDIY plasmids directly into the already constructed EEV (X). Alternatively, if the restriction enzyme sites accompanying the p*Tc*R provided SM were to complicate this step, the cloned genomic fragment could be employed as an intermediate traceable element recipient (XI). Later on through fragment subcloning (XII) the desired tagged EEV could be obtained.
To evaluate the feasibility of this methodology, the restriction enzyme site analysis of the predicted CL-Brener coding sequences was repeated. Apart from focusing only on restriction enzyme sites that could be used with those in pDIY vectors, the precise location of each detected site required for in frame insertion was considered. Roughly 89.7 % of the sequences contain at least one restriction site that could be used for the insertion of at least one pDIY encoded traceable element. Again, the practical feasibility has to be evaluated for each particular gene. Given that pDIY vectors can be used in different organisms the analysis was additionally performed on the predicted coding sequences of *Trypanosoma brucei* TREU 927 (12) and *Leishmania major* Friedlin (13) genomic reference strains and *T. brucei* Lister 427 strain. In these data sets respectively 87.4 %, 97.2 % and 90.5 % of the sequences contain adequate restriction sites. The extended results are available upon request.

5. Construction of EEVs combining p*Tc*R and pDIY vectors

There are various ways in which to combine p*Tc*R and pDIY vectors into EEVs. In general placing the coding sequence of pDIY downstream from the HX1 sequence of p*Tc*R-HG and then incorporating the resulting intergenic and coding sequences into a p*Tc*R-GA represents a straightforward methodology. The first step can be performed producing the coding sequence as a XbaI-XhoI fragment and inserting it into p*Tc*R-HG Neo + (for example) derived from dam+ bacteria and digested with the same enzymes. A BglII-MfeI fragment obtained from the resulting intermediate vector can be ligated into p*Tc*R-GA Neo + (among others) prepared with BglII and EcoRI. In this simple example, many restriction enzymes sites might become duplicated. However performing appropriate pDIY vector transformations in advance or employing adequate combinations of + and - p*Tc*R variants can help remove sites which might be needed for later additional cloning steps.

6. Suggested initial antibiotic concentrations.

		Construct		
**Marker**	**Antibiotic**	**pTREXL**	**pTEXL**	**p*Tc*R**
**ble**	Phleomycin	250	250	250
**BSD**	Blasticidin S	50	50	10

**Hyg**	Hygromycin B	500	250	200
**Neo**	Geneticin (G418)	500	100	100

Table S9: Initial antibiotic concentrations employed to select de epimastigotes transfected with the indicated constructs. The concentrations can be used as general guidelines for the selection of transgenic parasites.

7. References

1. Kelly, J.M., Ward, H.M., Miles, M.A. and Kendall, G. (1992) A shuttle vector which facilitates the expression of transfected genes in Trypanosoma cruzi and Leishmania. *Nucleic Acids Res*, 20, 3963-3969.
2. Martinez-Calvillo, S., Lopez, I. and Hernandez, R. (1997) pRIBOTEX expression vector: a pTEX derivative for a rapid selection of Trypanosoma cruzi transfectants. *Gene*, 199, 71-76.
3. Vazquez, M.P. and Levin, M.J. (1999) Functional analysis of the intergenic regions of TcP2beta gene loci allowed the construction of an improved Trypanosoma cruzi expression vector. *Gene*, 239, 217-225.
4. Taylor, M.C. and Kelly, J.M. (2006) pTcINDEX: a stable tetracycline-regulated expression vector for Trypanosoma cruzi. *BMC Biotechnol*, 6, 32.
5. Batista, M., Marchini, F.K., Celedon, P.A., Fragoso, S.P., Probst, C.M., Preti, H., Ozaki, L.S., Buck, G.A., Goldenberg, S. and Krieger, M.A. (2010) A high-throughput cloning system for reverse genetics in Trypanosoma cruzi. *BMC Microbiol*, 10, 259.
6. Xu, D., Brandan, C.P., Basombrio, M.A. and Tarleton, R.L. (2009) Evaluation of high efficiency gene knockout strategies for Trypanosoma cruzi. *BMC Microbiol*, 9, 90.
7. Evans, D.H., Willer, D.O. and Yao, X.D. (2009) DNA Joining Method. United States patent US 7575860.
8. Hamilton, M.D., Nuara, A.A., Gammon, D.B., Buller, R.M. and Evans, D.H. (2007) Duplex strand joining reactions catalyzed by vaccinia virus DNA polymerase. *Nucleic Acids Res*, 35, 143-151.
9. Chesnut, J.D., Carrino, J., Leong, L., Madden, K., Gleeson, M.A., Fan, J., Brasch, M.A., Cheo, D., Hartley, J.L., Byrd, D.R.N. *et al.* (2007) Methods and composition for synthesis of nucleic acid molecules using multiple recognition sites., United States patent US 7198924.
10. El-Sayed, N.M., Myler, P.J., Bartholomeu, D.C., Nilsson, D., Aggarwal, G., Tran, A.N., Ghedin, E., Worthey, E.A., Delcher, A.L., Blandin, G. *et al.* (2005) The genome sequence of Trypanosoma cruzi, etiologic agent of Chagas disease. *Science*, 309, 409-415.
11. Nozaki, T. and Cross, G.A. (1994) Functional complementation of glycoprotein 72 in a Trypanosoma cruzi glycoprotein 72 null mutant. *Mol Biochem Parasitol*, 67, 91-102.
12. Berriman, M., Ghedin, E., Hertz-Fowler, C., Blandin, G., Renauld, H., Bartholomeu, D.C., Lennard, N.J., Caler, E., Hamlin, N.E., Haas, B. *et al.* (2005) The genome of the African trypanosome Trypanosoma brucei. *Science*, 309, 416-422.
13. Ivens, A.C., Peacock, C.S., Worthey, E.A., Murphy, L., Aggarwal, G., Berriman, M., Sisk, E., Rajandream, M.A., Adlem, E., Aert, R. *et al.* (2005) The genome of the kinetoplastid parasite, Leishmania major. *Science*, 309, 436-442
